# Prevalence and Prognostic Value of Malnutrition Among Elderly Cancer Patients Using Three Scoring Systems

**DOI:** 10.3389/fnut.2021.738550

**Published:** 2021-10-11

**Authors:** Qi Zhang, Liang Qian, Tong Liu, Jia-Shan Ding, Xi Zhang, Meng-Meng Song, Zi-Wen Wang, Yi-Zhong Ge, Chun-Lei Hu, Xiang-Rui Li, Meng Tang, Kun-Hua Wang, Rocco Barazzoni, Chun-Hua Song, Hong-Xia Xu, Han-Ping Shi, Investigation on Nutrition Status and Its Clinical Outcome of Common Cancers (INSCOC) Group

**Affiliations:** ^1^Department of Gastrointestinal Surgery, Beijing Shijitan Hospital, Capital Medical University, Beijing, China; ^2^Department of Clinical Nutrition, Beijing Shijitan Hospital, Capital Medical University, Beijing, China; ^3^Beijing International Science and Technology Cooperation Base for Cancer Metabolism and Nutrition, Beijing, China; ^4^Graduate school, Capital Medical University, Beijing, China; ^5^Department of Obstetrics and Gynecology, Hangzhou Women's hospital, Hangzhou, China; ^6^Department of Obstetrics and Gynecology, Hangzhou Maternal and ChildHealth Hospital, Hangzhou, China; ^7^Department of Obstetrics and Gynecology, Hangzhou First People92s Hospital Qianjiang, Hangzhou, China; ^8^Department of Obstetrics and Gynecology, the First Affiliated Hospital of Nanchang University, Nanchang, China; ^9^The Second Affiliated Hospital and Yuying Children's Hospital of Wenzhou Medical University, Wenzhou, China; ^10^Department of Gastrointestinal Surgery, Institute of Gastroenterology, the First Affiliated Hospital of Kunming Medical University, Kunming, China; ^11^Department of Medical, Surgical and Health Sciences, University of Trieste, Trieste, Italy; ^12^Department of Epidemiology, College of Public Health, Zhengzhou University, Zhengzhou, China; ^13^Department of Clinical Nutrition, Daping Hospital, Army Medical University, Chongqing, China

**Keywords:** elderly patients, malnutrition index, cancer, prognostic, NRI

## Abstract

**Background:** Malnutrition is common in patients with cancer and is associated with adverse outcomes, but few data exist in elderly patients. The aim of this study was to report the prevalence of malnutrition using three different scoring systems and to examine the possible clinical relationship and prognostic consequence of malnutrition in elderly patients with cancer.

**Methods:** Nutritional status was assessed by using controlling nutritional status (CONUT), the prognostic nutritional index (PNI), and the nutritional risk index (NRI). Quality-of-life (Qol) was assessed during admission by using the European Organization for Research and Treatment of Cancer Quality of Life Questionnaire C-30. Performance status (PS) was assessed by using the Eastern Cooperative Oncology Group (ECOG) classification. The relationship between nutritional status and overall survival and Qol were examined.

**Results:** Data were available for 1,494 elderly patients with cancer (63.65% male), the mean age was 70.76 years. According to the CONUT, NRI, and PNI, 55.02, 58.70, and 11.65% patients were diagnosed with malnutrition, respectively. Worse nutritional status was related to older, lower BMI, lower hand grip strength, and more advanced tumor stage. All malnutrition indexes were correlated with each other (CONUT vs. PNI, *r* = −0.657; CONUT vs. NRI scores, *r* = −0.672; PNI vs. NRI scores, *r* = 0.716, all *P* < 0.001). During a median follow-up of 43.1 months, 692 (46.32%) patients died. For patients malnourished, the incidence rate (events-per-1,000person-years) was as follows: CONUT (254.18), PNI (429.91), and NRI (261.87). Malnutrition was associated with increased risk for all-cause mortality (adjust HR [95%CI] for CONUT: 1.09 [1.05–1.13], *P* < 0.001; PNI: 0.98[0.97–0.99], *P* < 0.001; NRI: 0.98 [0.98–0.99], *P* < 0.001). All malnutrition indexes improved the predictive ability of the TNM classification system for all-cause mortality. Deterioration of nutritional status was associated with deterioration in Qol parameters and immunotherapeutic response (*P* < 0.001).

**Conclusions:** Malnutrition was prevalent in elderly patients with cancer, regardless of the assessment tools used, and associated with lower Qol and the immunotherapy response.

## Introduction

Cancer is a devastating disease characterized by a poor prognosis, mainly in elderly patients. Clinical interventions for cancer have changed significantly in the past years. Although treatment options for patients with cancer have increased in the recent past, the prognosis remains relatively poor for elderly cancer patients. Malnutrition is common in elderly cancer patients; however, it is commonly ignored in routine clinical care (1). Changes in nutritional status and/or deterioration of the performance status (PS) are correlated with increased risk of acute toxicity, reduced therapy response, and shorter survival following anticancer treatment (2). Several studies have reported the importance of the nutritional status in elderly patients with cancer. Identifying high-risk patients based on modifiable clinical characteristics, such as nutritional status, is essential to recommend interventions targeting these variables to improve clinical outcomes and reduce health costs (2).

A previous study reports that malnutrition is an impairment poor prognostic factor in elderly patients with cancer (3). The death of several patients with cancer can be attributed to malnutrition rather than cancer itself (4). Malnutrition is an important factor in anticancer treatment and is reported in patients with various body weights and body mass indexes (BMI), independent from adiposity (5). Malnutrition can easily be alleviated; thus physicians can effectively manage it in various diseases (6). Screening patients with cancer for malnutrition can identify patients who can benefit from tailored intervention to prevent and treat malnutrition, improve prognosis and the quality of life (Qol) (7). European Society of Clinical Nutrition and Metabolism (ESPEN) guidelines recommend that all patients should be screened regularly for the presence or risk of malnutrition (8). Several malnutrition scoring indexes have been developed to evaluate immunocompetence and nutritional conditions for patients, such as the controlling nutritional status (CONUT) index (9), the prognostic nutritional index (PNI), and the nutritional risk index (NRI). Data obtained using these malnutrition indexes are quantitative and can be obtained by routine blood testing in various institutions.

Currently, findings on the interaction between these assessment tools and their comparative use for the prediction of clinical outcomes in elderly patients with cancer are limited. The prevalence of malnutrition varies depending on the assessment tools used. The aim of the present study was to explore the prevalence of malnutrition using three different scoring systems and to evaluate the possible clinical relationship and prognostic value of malnutrition in elderly cancer patients.

## Method and Participants

### Study Population

This is a prospective cohort study based on the investigation on nutrition status and its clinical outcome of common cancers (INSCOC) cohort in China. The trial was registered at http://www.chictr.org.cn under the registration number ChiCTR1800020329. Data were collected prospectively from multicenter across China. The design, methods, and development of the INSCOC study were as described previously (10, 11). All patients included in the INSCOC cohort were diagnosed with solid tumors and were 18 yr old or older. Patients were examined through a survey before undergoing cancer treatment (surgery, chemotherapy, radiotherapy, or other treatments). Participants were enrolled in this cohort as only inpatients, requiring an inpatient stay >48 h. Patients who could not communicate and/or were unable to provide verbal consent were excluded from the study. The study was conducted following the principles outlined in the Declaration of Helsinki and was approved by the ethical committee from each local center. Written/verbal informed consent for using clinical data without revealing personal information was obtained from all participants. Patients aged 65 years or older were included in the current secondary analysis. Patients who had no records of height, weight, scrum albumin, cholesterol, or lymphocyte count were excluded from the study. Patients with clinical evidence of active infection and the presence of immunologic disease were also excluded from the study. No patient had suspected or documented bone marrow involvement. A flow diagram for study subject screening and grouping is shown in Supplementary Figure 1.

### Demographics and Clinical Characteristics

Data on age, sex, primary cancer type, and tumor stage were obtained from the electronic medical record system. Body mass index (BMI), defined as the weight (kilograms) divided by the square of height (meters), was calculated for all patients. Patients were classified into four groups including underweight (<18.5 kg/m^2^), normal weight (18.5–23.9 kg/m^2^), overweight (24.0–28.0 kg/m^2^), and obese (>28 kg/m^2^). Chronic disease was defined as any previous history of hypertension, diabetes, chronic obstructive pulmonary disease, and chronic hepatitis, and information on the history of chronic disease was retrieved from the clinical history of the diagnoses recorded in patients notes. The clinical stage of cancer was evaluated by TNM staging based on the 8th AJCC TNM classification system.

Performance status was determined by the Eastern Cooperative Oncology Group (ECOG). Patients were classified in different categories ranging from grade 0 (fully active) to grade 5 (dead). ECOG grade 5 was excluded in the current study. Patient-generated subjective nutrition assessment (PG-SGA) was assessed and recorded by trained staff at baseline. Data on Qol were collected on the day of admission using the European Organization for Research and Treatment of Cancer Quality of Life Questionnaire (EORTCQLQ-C30 Version 3.0, Qol). The QLQ-C30 scale is a 30-item questionnaire comprising functional assessment (physical, role, emotional, social, and cognitive), symptom assessment (fatigue, nausea and vomiting, and pain), and global health and Qol assessment (dyspnea, insomnia, appetite loss, constipation, diarrhea, and financial difficulties) (12). Overall and subscale scores were calculated following specific guidelines (higher scores indicated better Qol).

### Malnutrition Assessment

We reassessed the nutritional status (using CONUT, PNI, and NRI) based on the data collected during the baseline. CONUT, PNI, and NRI indexes were calculated using the following formula:

CONUT: includes serum albumin level, total cholesterol level, and lymphocyte count (9); each parameter can be scored as 0, 1, or 2.

PNI: 10^*^serum albumin (g/dl) + 0.005^*^total lymphocyte count (mm^3^).

NRI: 1.489^*^serum albumin (g/l) + 41.7^*^ (weight in kilograms/ideal weight).

Venous blood sample (scrum albumin, total lymphocyte count, and cholesterol) was collected on the first day of admission after overnight fasting. All the measurements were analyzed at a central laboratory and standardized to avoid differences caused by location and/or scale of measurements between laboratories. Patients were classified into absent, mild (expect PNI), moderate, and severe malnutrition risk based on CONUT, PNI, and NRI indexes as shown in Supplementary Table 1 (13).

### Outcome and Follow-up

All-cause mortality was the primary endpoint in the current study. Patients were regularly followed up by telephone or outpatient visits to collect information on clinical outcomes. Overall survival was expressed in months and defined as the time from the date of admission until death or censored if alive at follow-up analysis (30 December 2019).

### Statistical Analysis

Demographic characteristics of the study population were described. Continuous data were expressed as mean and standard deviation (unless otherwise specified), and categorical data were expressed as a number and percentage (n, %). Independent students *t*-test or non-parametric tests were used to compare differences between groups. Multiple groups were compared by one-way ANOVA with an appropriate *post-hoc* test. Pearson chi-square test or Fisher's exact test was used for comparing proportions between the groups. Correlation between quantitative variables was explored through Pearson's correlation analysis. Venn diagrams were used to illustrate the relationship between the three malnutritional indexes. We selected covariates and potential confounders *a priori*, based on previous scientific knowledge (14). Variable was removed from the model where variables were highly intercorrelated (multicollinearity). Univariate and multivariate COX regression analyses were performed to evaluate hazard ratios (HRs) and 95% confidence intervals (CIs) of significant risk predictors based on over survival. A restricted cubic spline plot was used to explore the shape of the correlation between malnutrition index and clinical outcome. Kaplan–Meier curves and log-rank tests were used to present time-to-event data and compare survival between groups, respectively. Harrell C-index (15), continuous net reclassification improvement **(cNRI)** (16), integrated discrimination improvement **(IDI)** **(****17****)**, and time– area under the curve (AUC) were calculated to assess and compare the discrimination capacity of the three malnutrition indexes to predict mortality. A two-sided *p*-value of 0.05 was considered statistically significant. All statistical analyses were performed using R, version 4.0.2 software (R Foundation for Statistical Computing, Vienna, Austria).

## Results

### Patient Characteristics

A total of 1,494 elderly participants diagnosed with cancer were enrolled in the current study. Most patients were male (63.65%), and the mean age was 70.76 years. The prevalence of distant metastases was high (42.70%), and the most common cancer was digestive cancer (50.80%). All patients underwent radiotherapy, and 62.85% of the patients included in this study underwent systemic chemotherapy treatment. Hundred and nine (7.30%)patients received immunotherapy (PD-1/PD-L1). However, patients presented with low mean Qol, as indicated by mean QLQ-C30 score at 39.21 ± 4.79, showed a PS that indicated normal capability with independent daily activities (ECOG: 1.09 ± 0.80). Details on the baseline characteristics of this study are presented in Table 1.

**Table 1:** Baseline characteristics of the study population.

**Characteristics**	**Overall**
	***n* = 1,494**
**Demographic data**	
Age, years	70.76 (5.16)
Gender, male	951(63.65%)
BMI, kg/m^2^	22.58 (3.55)
Smoking, yes	696 (46.59%)
Alcohol, yes	289 (19.34%)
Comorbidities	
Absent	1,081 (72.36%)
Hypertension	337 (22.56%)
Others	76 (5.09%)
**Disease data**	
Tumor location	
Lung	458 (30.66%)
Digestive	759 (50.80%)
Other	277 (18.54%)
Tumor stage:	
I	147 (9.84%)
II	336 (22.49%)
III	373 (24.97%)
IV	638 (42.70%)
Chemotherapy, yes	939 (62.85%)
Radiotherapy, yes	1,494(100.00%)
Immunotherapy, yes	109 (7.30%)
Surgery, yes	357 (23.90%)
ECOG	1.09 (0.80)
**Laboratory data**	
Albumin, g/dl	3.80 (0.53)
Cholesterol, mg/dl	182.95 (60.94)
Lymphocyte, *10^9^/L	1.67 (1.37)
**Nutritional data**	
CONUT, as continuous	2.30 (2.21)
Category	
Absent	672 (44.98%)
Mild	602 (40.29%)
Moderate	193 (12.92%)
Severe	27 (1.81%)
PNI, as continuous	46.05 (9.90)
Category	
Absent	1,320 (88.35%)
Moderate	77 (5.15%)
Severe	97 (6.49%)
NRI, as continuous	96.96 (10.32)
Category	
Absent	617 (41.30%)
Mild	174 (11.65%)
Moderate	607 (40.63%)
Severe	96 (6.43%)
PG-SGA, as continuous	6.26 (4.48)
Category	
Absent	52 (3.48%)
Mild	503 (33.67%)
Moderate	549 (36.75%)
Severe	390 (26.10%)
HGS, kg	22.38 (8.93)
EORTCQLQ-C30	39.21 (4.79)
PN, yes	188 (12.58%)
EN, yes	146 (9.77%)

*Values are mean (SD) or n (%). BMI, body mass index; ECOG, Eastern Cooperative Oncology Group; CONUT, Controlling Nutritional Status score; PNI, prognostic nutritional index; NRI, nutritional risk index; PG-SGA, a patient-generated subjective global assessment; HGS, handgrip strength; EORTCQLQ-C30, European Organization for Research and Treatment of Cancer Quality of Life Questionnaire; PN, parenteral nutrition; EN, enteral nutrition*.

### Prevalence and Clinical Association of Malnutrition

**Figure 1:**
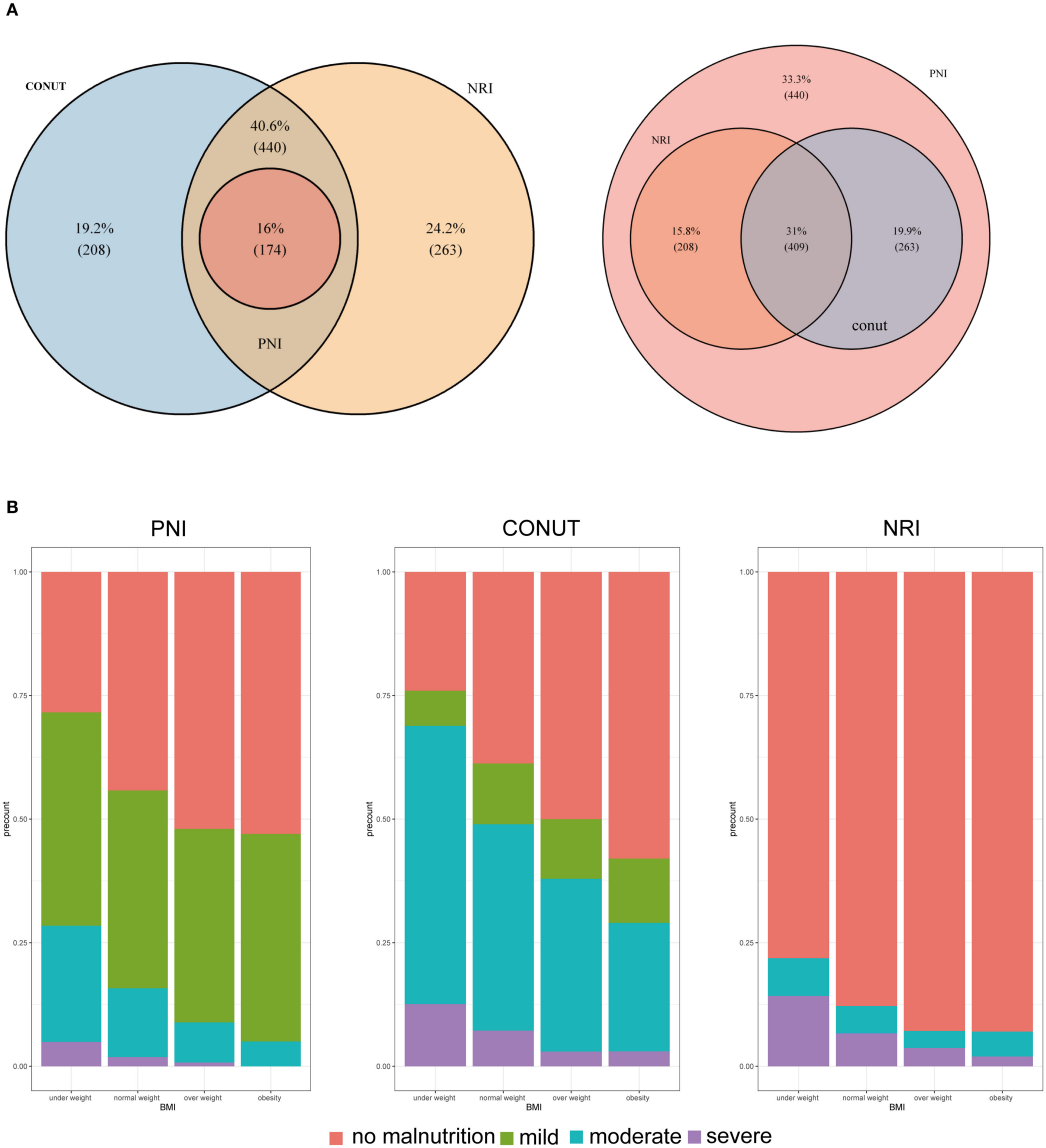
**(A)** Venn diagram. The numbers reported in each circle indicate the cumulative frequency of malnutrition (any degree [left] vs. no-malnourished [right]) according to each malnutrition index. **(B)** Percentage of malnutrition by subgroups of patients according to body mass index. CONUT: Controlling Nutritional Status score, PNI: prognostic nutritional index, NRI: nutritional risk index.

The proportion of patients with malnutrition varied from 55.02% with the CONUT to 58.70% with the NRI, and 11.65% with the PNI. Analysis using CONUT, NRI, and PNI indexes showed that 220 (14.73%), 703(47.60%), and 174 (11.65%) patients, respectively, had moderate to severe malnutrition. Patients with malnutrition determined by any of the three malnutrition indices were mainly older, had lower BMI, lower handgrip strength, and presented with more advanced tumor stage (Supplementary Tables 2–4). All malnutrition indices were correlated with each other (CONUT vs. PNI, *r* = −0.657, *P* < 0.001; CONUT vs. NRI scores, *r* = −0.672, *P* < 0.001; PNI vs. NRI scores, *r* =0.716, *P* < 0.001, Supplementary Figure 2), but showed a weak concordance (Supplementary Table 5). In addition, all malnutrition indices were weakly correlated with PG-SGA (*r* = 0.278 for CONUT; *r* = −0.173 for PNI, and *r* = −0.300 for NRI, all *P* < 0.001) and had low validity (AUC = 0.595 for CONUT, AUC = 0.545 for PNI, and AUC = 0.617 for NRI) and reliability (κ = 0.18 for CONUT, κ = 0.07 for PNI, and κ = 0.23 for NRI) compared with the PG-SGA (Supplementary Table 6). Notably, 174 (11.65%) patients were classified as having malnutrition by all of the three malnutrition indices, and 409 (27.38%) patients were not diagnosed with malnutrition based on the three scores (Figure 1A). Analysis of BMI showed that participants with lower BMIs had a higher prevalence of malnutrition compared with those with higher BMI (Figure 1B). Most patients with a BMI above 25 kg/m^2^ were also diagnosed with malnutrition by the three malnutrition indices: CONUT (159, 46.49%), NRI (169, 49.42%), and PNI (21, 6.14%).

### Malnutrition Indices and Mortality

**Figure 2:**
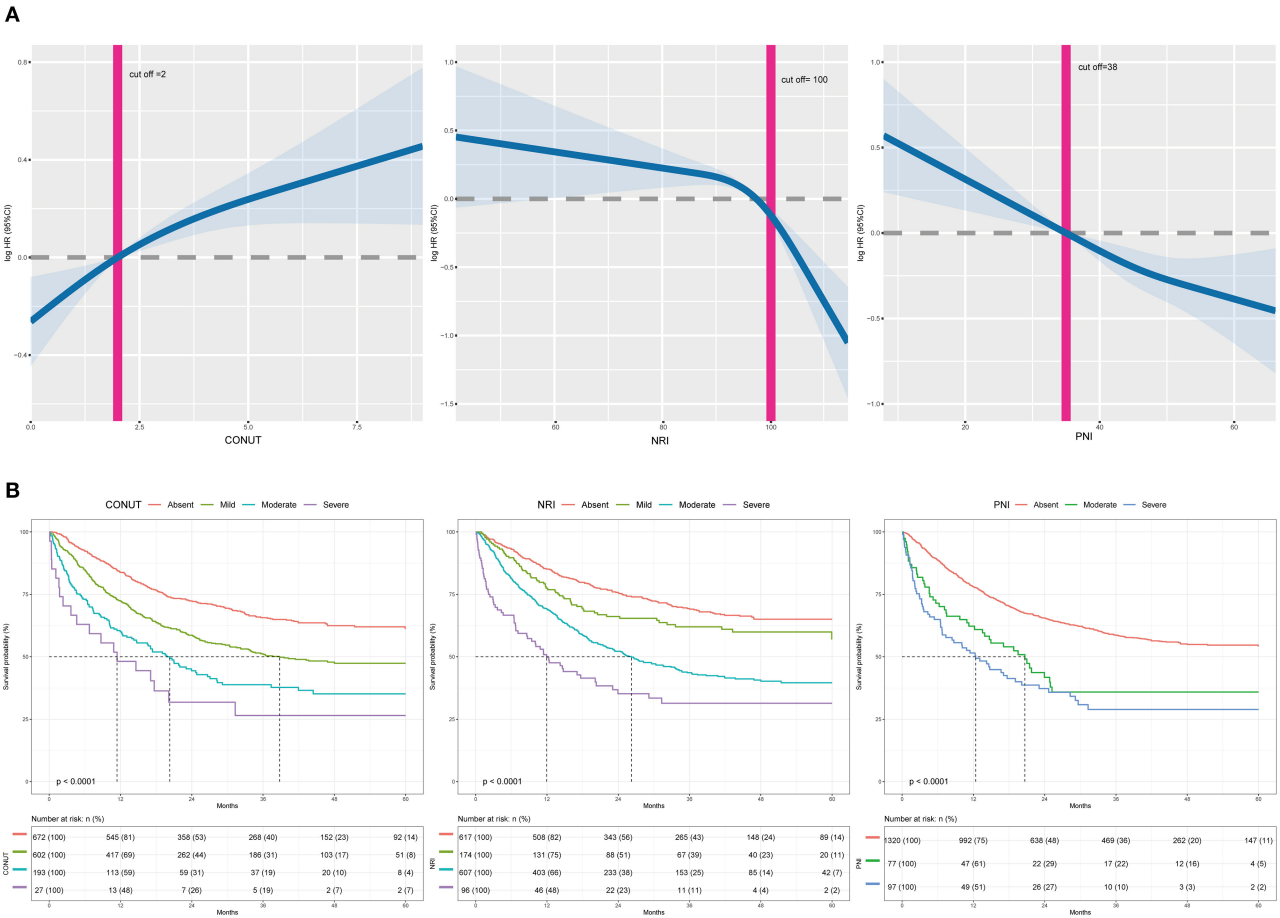
**(A)** The incidence of all-cause mortality is shown after adjusted for age, gender, BMI, comorbidities disease, smoking, alcohol, tumor location, tumor stage, chemotherapy, immunotherapy, surgery, parenteral nutrition intervention, enteral nutrition intervention, ECOG, handgrip strength, and EORTCQLQ-C30. For NRI, total cholesterol and lymphocyte count were adjusted additionally. For PNI, lymphocyte count was adjusted additionally. The *x*-axis shows the score of each malnutrition index. The curve shows the incidence, with 95% CI, of the estimates. **(B)** Kaplan-Meier curves for all-cause mortality by the category of each malnutrition index in elderly patients with cancer. CONUT, Controlling Nutritional Status score; PNI, prognostic nutritional index; NRI, nutritional risk index.

A total of 692 (46.32%) patients died within a median follow-up of 43.1 months. Univariable predictors of mortality for this study population are presented in Supplementary Table 7. Malnutrition status was correlated with a higher incidence of mortality regardless of the malnutrition index used. Incidence rates for the three indexes (events per 1,000 person-years) were as follows: CONUT (254.18), PNI (429.91), and NRI (261.87). Poor malnutrition status was correlated with poor OS, independent of whether the scores were analyzed as a continuous (Figure 2A) or a categorical variable (Table 2). Kaplan–Meier curves and adjusted curves were used to explore the relationship between malnutrition status and overall survival (Figure 2B and Supplementary Figure 3). Specific analysis by tumor location (lung, digestive, or other) is presented in Supplementary Table 8. After exclusion of 6 months mortality (209, 13.99%), a significant correlation between malnutrition status and OS was observed (Supplementary Table 9).

**Table 2:** Cox proportional analyses of malnutrition indexes to predict all-cause mortality for elderly patients with cancer.

	**Crude HR(95%CI)**	***P*-value**	**Adjusted HR (95% CI)^**a**^**	***P*-value**	**Adjusted HR(95%CI)^**b**^**	***P*-value**
CONUT, as continuous	1.16 (1.12–1.19)	<0.001	1.16 (1.12–1.19)	<0.001	1.09 (1.05–1.13)	<0.001
Category						
Absent	Ref					
Mild	1.64 (1.37–1.95)	<0.001	1.52 (1.28–1.82)	<0.001	1.34 (1.12–1.61)	0.002
Moderate	2.48 (1.97–3.11)	<0.001	2.05 (1.62–2.59)	<0.001	1.72 (1.34–2.20)	<0.001
Severe	3.48 (2.18–5.56)	<0.001	2.85 (1.78–4.58)	<0.001	1.89 (1.14–3.13)	0.014
PNI, as continuous	0.97 (0.96–0.98)	<0.001	0.97 (0.97–0.98)	<0.001	0.98 (0.97–0.99)	<0.001
Category						
Absent	Ref					
Moderate	1.91 (1.41–2.59)	<0.001	1.72 (1.26–2.33)	<0.001	1.60 (1.17–2.19)	0.004
Severe	2.58 (1.99–3.34)	<0.001	2.19 (1.68–2.85)	<0.001	2.08 (1.58–2.73)	<0.001
NRI, as continuous	0.98 (0.97–0.98)	<0.001	0.98 (0.97–0.98)	<0.001	0.98 (0.98–0.99)	<0.001
Category						
Absent	Ref					
Mild	1.34 (1.01–1.78)	0.044	1.29 (0.97–1.71)	0.083	1.29 (0.97–1.71)	0.084
Moderate	2.23 (1.85–2.67)	<0.001	1.98 (1.64–2.38)	<0.001	1.74 (1.44–2.10)	<0.001
Severe	3.70 (2.77–4.95)	<0.001	3.05 (2.26–4.10)	<0.001	2.67 (1.95–3.64)	<0.001

*a: Adjusted by age, gender, BMI. b: Adjusted by age, gender, BMI, comorbidities disease, smoking, alcohol, tumor location, tumor stage, chemotherapy, immunotherapy, surgery, parenteral nutrition intervention, enteral nutrition intervention, ECOG, handgrip strength, EORTCQLQ-C30. For NRI, total cholesterol and lymphocyte count were adjusted additionally. For PNI, lymphocyte count was adjusted additionally. HR, hazard ratio; CI, confidence interval; CONUT, Controlling Nutritional Status score; PNI, prognostic nutritional index; NRI, nutritional risk index*.

A comparison of the malnutrition index is summarized in Table 3. C-index analyses were performed to compare the clinical implications of the three malnutrition indices. NRI showed the highest C-index for OS (0.641, 95%CI 0.62–0.66), followed by CONUT (0.61, 95% CI0.59–0.63), and PNI (0.56, 0.53–0.58). In addition, NRI exhibited a significantly higher AUC value compared with the other two malnutrition indices (Supplementary Figure 4). However, NRI score performance was similar with CONUT and PNI indexes at predicting OS, as shown by the discrimination index values (Supplementary Figure 5). Findings on OS prediction showed that each of the three malnutrition indices had a significant prognostic value on the TNM classification system. NRI index showed the highest incremental value.

**Table 3:** Comparative analysis of the discrimination and model performance of each malnutrition index for all-cause mortality.

	**CONUT**		**NRI**		**PNI**	
C-index	0.607(0.585–0.630)		0.641(0.619–0.663)		0.557(0.534–0.581)	
	CONUT vs. NRI		CONUT vs. PNI		NRI vs. PNI	
	Difference	*P*-value	Difference	*P*-value e	Difference	*P*-value e
cNRI	−0.025	0.553	−0.081	0.058	−0.086	0.06
IDI	−0.01	0.368	0.029	0.11	−0.019	0.214
Model	C–index	*P*-value	cNRI	*P*-value	IDI	*P*-value
TNM stage	0.713(0.693–0.732)	Ref	Ref		Ref	
TNM stage +CONUT	0.731(0.712–0.750)	<0.001	0.139	0.016	0.025	<0.001
TNM stage +NRI	0.732(0.713–0.751)	<0.001	0.237	0.006	0.018	0.004
TNM stage +PNI	0.723(0.703–0.742)	<0.001	0.196	0.01	0.015	0.004

*CONUT, Controlling Nutritional Status score; PNI, prognostic nutritional index; NRI, nutritional risk index*.

### Relationship Between Malnutrition and Qol and Immunotherapy

The relationship between malnutrition status and symptom components of the Qol is presented in Supplementary Tables 10–12. Deterioration of nutritional status was independently associated with worsening of most of the symptoms (PF, RF, CF, SF, QL, FA, NV, PA, DY, SL, AP, FI, *P* < 0.05). The findings showed a deterioration in PF across malnutrition categories, despite having good PS (ECOG < 2). A similar pattern was observed with RF, CF, SF, QL, FA, and AP (*P* < 0.05 Supplementary Tables 13–15). Patients were stratified to explore the correlation between malnutrition status and immunotherapy outcomes. The findings showed that patients with a poor malnutrition status who underwent immunotherapy had a poor prognosis (Supplementary Table 16 and Supplementary Figure 6).

## Discussion

The current study included elderly cancer patients. Out of the total patients included in the study, patients classified as malnourished ranged between 11.65 and 58.70% based on different screening tools. Moderate to severe malnutrition, dependent upon the tool used, ranged from 11.65 to 47.60%. Notably, malnutrition was prevalent even in overweight or obese patients. Most patients with a BMI > 24 were diagnosed with malnutrition (47.46% with CONUT, 7.03% with PNI, and 48.24% with NRI). Malnutrition was prevalent in elderly patients with cancer, and it is associated with all-cause mortality regardless of the malnutrition index used tumor types, and other risk factors. Moreover, malnutrition was associated with functional decline and was correlated with deterioration of Qol in elderly patients with cancer. In addition, changes in nutritional status were correlated with the prognosis of immunotherapy.

A previous study had reported that the nutritional assessment tool is associated with poor outcomes in elderly patients with cancer (3). Notably, only few studies have fully explored the prevalence and prognostic value of malnutrition index in elderly patients with cancer (18). The current study comprises a growing elderly population, and malnutrition is highly prevalent (19). Notably, aging and lack of physical activity are risk factors for malnutrition in the elderly (20). Malnutrition assessment should be carried out in inpatient facilities and should be recommended as a necessity to increase anticancer treatment efficacy. Although being underweight is a criterion of undernutrition, being overweight or obese does not protect against malnutrition (21). A previous study had shown that 20% of elderly cancer patients with malnutrition were obese. BMI was found to be a less valid screening tool for determining malnutrition in elderly patients with cancer (22). In addition, the use of individualized nutritional support is recommended in elderly patients with cancer who are already malnourished or are at a risk of becoming malnourished (23). These patients are predisposed to age-related sarcopenia and reduced gastrointestinal absorption (24).

With more sophisticated use of nutritional intervention, early identification of malnutrition is important, especially with the emphasis on patient-centered care (25). Severe malnutrition scoring indexes, such as the CONUT, PNI, and NRI have been developed and validated in the past to help in the identification of inflammatory or malnourished patients at the risk of complications (26). Serum albumin can accurately reflect both nutritional and inflammatory status and is independently correlated with survival in patients with colorectal cancer (27). NRI is different from CONUT and PNI indexes as it includes both anthropometric factors and serum markers. In this study, NRI showed the highest incremental value in predicting mortality risk compared with CONUT and PNI indexes. However, these three indices were weakly correlated with PG-SGA and had low validity and reliability compared with the PG-SGA. One possible explanation is that PG-SGA predominantly relies on subjective answers of patients, but the nutritional index used in this study considers only objective variables. Subjective variables often entail patient participation, which may be time consuming and affected by patient perceptions (28). Particularly, concordance among scores for identifying degrees of malnutrition was rather poor, suggesting that they are not interchangeable (13).

Elderly patients with cancer exhibit several complications owing to their higher risk of malnutrition. Malnourished patients present with poor PS due to fatigue, loss of control and independency, high level of systemic inflammation, and ultimately impairing Qol (29, 30). Moreover, the traditional outcome of OS may be inappropriate in elderly patients (31). Previous studies report that poor nutritional status is correlated with poor Qol, for patients treated with curative and palliative interventions (32). A multicenter study comprising of 1,027 advanced cancer patients reported that malnutrition, PS (ECOG), and systemic inflammation were significantly correlated with poor Qol (33). The findings of the current study showed that malnutrition is correlated with poor Qol, even in patients with good PS. A high level of malnutrition was correlated with high levels of systemic inflammation. Meanwhile, several proinflammatory cytokines, such as TNF alpha, IL 6, and hormones would be produced by the tumor directly or systemically in response to the tumor, which had also been reported in the pathogenesis of malnutrition (21, 34). Malnutrition is a common cause of secondary immunologic dysfunction, as it results in a decrease in the total lymphocyte count (13, 35). Lymphocytes, mainly CD4+ and CD8+ T cells, play important roles in the immune response to immunotherapy (36). Consistent with this finding, the current study showed that patients with poor nutritional status had poor OS after receive immunotherapy.

The present study had several limitations. Data included in the study were limited to Chinese patients. Differences in genetic background, lifestyle, and diets may contribute to the differences in the Chinese population compared with other populations. Additionally, there are some potential selection bias, information bias, and residual confounding in our study in that we are not able to conduct a comprehensive geriatric assessment in elderly patients with cancer (37, 38). Some geriatric predictors such as depression, dementia, frailty, and functional impairment were not collected at the beginning of the study. Meanwhile, all of the patients in this study were inpatients and nutritional assessment was conducted only at admission. The study did not explore changes in nutritional status over time and their relationship with Qol and mortality. However, to the best of our knowledge, the current study is the first to explore the relationship between malnutrition index, Qol, and all-cause mortality in a large number of elderly patients with cancer. The study population was representative of the general Chinese elderly patients diagnosed with cancer.

In summary, malnutrition was prevalent in elderly cancer patients, regardless of the assessment tools used. Malnutrition was correlated with lower Qol and poor immunotherapy response. Moreover, malnutrition is a potential independent prognostic factor in elderly patients with cancer. Therefore, assessment of nutritional status may be important for the management of elderly patients with cancer. Optimizing Qol and prolonging the survival time is the central tenet of cancer care in elderly cancer patients.

## Data Availability Statement

The raw data supporting the conclusions of this article will be made available by the authors, without undue reservation.

## Ethics Statement

The studies involving human participants were reviewed and approved by ethics committee of army medical center of PLA. The patients/participants provided their written informed consent to participate in this study.

## Author Contributions

H-PS had full access to all the data in the study and takes responsibility for the integrity of the data and the accuracy of the data analysis. H-PS and QZ conception and design. H-PS financial support. H-PS administrative support. X-RL, XZ, C-HS, RB, Y-ZG, J-SD, LQ, MT, C-LH, K-HW, H-XX, TL, and INSCOC group provision of study materials or patients. MT, XZ, and QZ collection and assembly of data: QZ, LQ, TL, J-SD, and Z-WW data analysis and interpretation. QZ manuscript writing. All authors contributed to the article and approved the submitted version.

## Funding

This study was supported by the National Key Research and Development Program: the key technology of palliative care and nursing for cancer patients (2017YFC1309200).

## Conflict of Interest

The authors declare that the research was conducted in the absence of any commercial or financial relationships that could be construed as a potential conflict of interest.

## Publisher's Note

All claims expressed in this article are solely those of the authors and do not necessarily represent those of their affiliated organizations, or those of the publisher, the editors and the reviewers. Any product that may be evaluated in this article, or claim that may be made by its manufacturer, is not guaranteed or endorsed by the publisher.

## Supplementary Material

Supplementary Table 1 Procedures for the evaluation of each nutritional index.

Supplementary Table 2 Baseline Characteristics of the Study Population stratified by CONUT.

Supplementary Table 3 Baseline Characteristics of the Study Population stratified by NRI.

Supplementary Table 4 Baseline Characteristics of the Study Population stratified by PNI.

Supplementary Table 5 Kappa agreement for CONUT, PNI, NRI index.

Supplementary Table 6 Sensitivity, specificity, positive predictive value and negative predictive value for CONUT, PNI, NRI index compared with PG-SGA.

Supplementary Table 7 Univariable Cox regression analyses of factors predicting all-cause mortality.

Supplementary Table 8 Multivariate Cox proportional hazards analyses of malnutrition indexes to predict all-cause mortality according to the location of cancer.

Supplementary Table 9 Multivariate Cox proportional hazards analyses of malnutrition indexes to predict all-cause mortality according to exclude patients died within 6 months.

Supplementary Table 10 Each parameter of EORTCQLQ–C30 stratified by CONUT.

Supplementary Table 11 Each parameter of EORTCQLQ–C30 stratified by NRI.

Supplementary Table 12 Each parameter of EORTCQLQ–C30 stratified by PNI.

Supplementary Table 13 Each parameter of EORTCQLQ–C30 (with ECOG less than 2) stratified by CONUT.

Supplementary Table 14 Each parameter of EORTCQLQ–C30 (with ECOG less than 2) stratified by NRI.

Supplementary Table 15 Each parameter of EORTCQLQ–C30 (with ECOG less than 2) stratified by PNI.

Supplementary Table 16 Hazard risk for all-cause mortality in elder patients treated with immunotherapy.

Supplementary Figure 1 Flow chart.

Supplementary Figure 2 Correlation analysis of clinical parameters.

Supplementary Figure 3 Adjusted Kaplan-Meier curves for all-cause mortality by the category of each malnutrition index in elderly patients with cancer. CONUT, Controlling Nutritional Status score; PNI, prognostic nutritional index; NRI, nutritional risk index. The value was adjusted for age, gender, body mass index, hypertension, other comorbidities disease, smoking, alcohol, tumor location, tumor stage, chemotherapy, immunotherapy, surgery, parenteral nutrition intervention, enteral nutrition intervention, ECOG, PG-SGA, handgrip strength, EORTCQLQ-C30. For NRI, total cholesterol and lymphocyte count were adjusted additionally. For PNI, lymphocyte count was adjusted additionally.

Supplementary Figure 4 Time-dependent area under the curve (AUC) by the three malnutrition indexes.

Supplementary Figure 5 Plot to graphically display Integrated Discrimination Improvement (IDI), continuous Net Reclassification Improvement (NRI), and median improvement, for the additional value of malnutrition scores to TNM stage as assessed by the paired difference of risk scores.

Supplementary Figure 6 Kaplan-Meier curves for all-cause mortality by the nutritional status in elderly patients with cancer treatment with immunotherapy.
