# Supplementary material for: Prevalence and Prognostic Value of Malnutrition Among Elderly Cancer Patients Using Three Scoring Systems
Source: Front Nutr. 2021 Oct 11;8:738550. doi: 10.3389/fnut.2021.738550 (PMC8544751; doi:10.3389/fnut.2021.738550)

Supplementary table 1. Procedures for the evaluation of each nutritional index.

Nutritional Indices	Risk of Malnutrition			
	Absent	Mild	Moderate	Severe
CONUT	0-1	2-4	5-8	9-12
Albumin, g/ dL(score)	≥3.5(0)	3.0-3.4(2)	2.5-2.9(4)	<2.5(6)
Total cholesterol, mmol/L (score)	≥180(0)	140-199(1)	100-139(2)	<100(3)
Lymphocyte count, $*10^9$/L(score)	≥1.60(0)	1.20-1.59(1)	0.80-1.19(2)	<0.80(3)
NRI	≥100	97.50-99.99	83.50-97.49	<83.50
PNI	>38	-	35-38	<35

CONUT: Controlling Nutritional Status score, PNI: prognostic nutritional index, NRI: nutritional risk index

Supplementary table 2. Baseline Characteristics of the Study Population stratified by CONUT.

COUNT	Overall n=1494	Absent n=672	Mild malnutrition n=602	Moderate malnutrition n=193	Severe malnutrition n=27	*P*-value
Age, years	70.76(5.16)	70.07(4.84)	71.04(5.21)	72.13(5.43)	71.67(7.18)	0.001
Gender, male	951(63.65%)	397(59.08%)	397(65.95%)	137(70.98%)	20(74.07%)	0.004
BMI, kg/m^2	22.58(3.55)	23.15(3.42)	22.47(3.63)	21.33(3.32)	19.71(2.97)	0.001
Smoking, yes	696(46.59%)	304(45.24%)	283(47.01%)	91(47.15%)	18(66.67%)	0.177
Alcohol, yes	289(19.34%)	113(16.82%)	132(21.93%)	39(20.21%)	5(18.52%)	0.143
Comorbidities						
Absent	1081(72.36%)	499(74.26%)	428(71.10%)	134(69.43%)	20(74.07%)	
Hypertension	337(22.56%)	155(23.07%)	130(21.59%)	48(24.87%)	4(14.81%)	
Others	76(5.09%)	18(2.68%)	44(7.31%)	11(5.70%)	3(11.11%)	
Tumor location						0.005
Lung	458(30.66%)	198(29.46%)	198(32.89%)	53(27.46%)	9(33.33%)	
Digestive	759(50.80%)	324(48.21%)	303(50.33%)	118(61.14%)	14(51.85%)	
Other	277(18.54%)	150(22.32%)	101(16.78%)	22(11.40%)	4(14.81%)	
Tumor stage:						<0.001
I	147(9.84%)	84(12.50%)	50(8.31%)	13(6.74%)	0(0.00%)	
II	336(22.49%)	167(24.85%)	122(20.27%)	40(20.73%)	7(25.93%)	
III	373(24.97%)	176(26.19%)	149(24.75%)	43(22.28%)	5(18.52%)	
IV	638(42.70%)	245(36.46%)	281(46.68%)	97(50.26%)	15(55.56%)	
Chemotherapy, yes	939(62.85%)	420(62.50%)	399(66.28%)	105(54.40%)	15(55.56%)	0.023
Immunotherapy, yes	109(7.30%)	47(6.99%)	49(8.14%)	13(6.74%)	0(0.00%)	0.483
Surgery, yes	357(23.90%)	171(25.45%)	122(20.27%)	57(29.53%)	7(25.93%)	0.034
ECOG	1.09(0.80)	0.95(0.66)	1.13(0.83)	1.35(0.88)	1.78(1.28)	<0.001
Albumin, g/dl	3.80(0.53)	4.08(0.33)	3.78(0.42)	3.07(0.48)	2.54(0.34)	<0.001
Cholesterol, mg/dl	182.95(60.94)	204.82(50.00)	175.02(67.04)	142.14(40.34)	106.99(25.03)	<0.001
Lymphocyte, 10^9/L	1.67(1.37)	2.08(1.28)	1.42(1.53)	1.12(0.50)	0.85(0.40)	<0.001
CONUT, as continuous	2.30(2.21)	0.49(0.50)	2.77(0.79)	6.10(1.02)	9.56(0.85)	<0.001
Category						
Absent	672(44.98%)	672(100.00%)	0(0.00%)	0(0.00%)	0(0.00%)	
Mild	602(40.29%)	0(0.00%)	602(100.00%)	0(0.00%)	0(0.00%)	
Moderate	193(12.92%)	0(0.00%)	0(0.00%)	193(100.00%)	0(0.00%)	
Severe	27(1.81%)	0(0.00%)	0(0.00%)	0(0.00%)	27(100.00%)	
PNI, as continuous	46.05(9.90)	51.16(7.10)	44.97(8.12)	34.26(9.19)	27.26(8.75)	<0.001
Category						
Absent	1320(88.35%)	672(100.00%)	579(96.18%)	69(35.75%)	0(0.00%)	
Moderate	77(5.15%)	0(0.00%)	22(3.65%)	55(28.50%)	0(0.00%)	
Severe	97(6.49%)	0(0.00%)	1(0.17%)	69(35.75%)	27(100.00%)	
NRI, as continuous	96.96(10.32)	101.73(5.34)	97.10(6.85)	83.02(14.57)	74.54(13.20)	<0.001
Category						
Absent	617(41.30%)	409(60.86%)	202(33.55%)	6(3.11%)	0(0.00%)	
Mild	174(11.65%)	101(15.03%)	70(11.63%)	3(1.55%)	0(0.00%)	
Moderate	607(40.63%)	162(24.11%)	323(53.65%)	117(60.62%)	5(18.52%)	
Severe	96(6.43%)	0(0.00%)	7(1.16%)	67(34.72%)	22(81.48%)	
PG-SGA, as continuous	6.26(4.48)	5.10(3.70)	6.67(4.58)	8.41(5.03)	10.56(5.77)	<0.001
Category						
Absent	52(3.48%)	38(5.65%)	11(1.83%)	3(1.55%)	0(0.00%)	
Mild	503(33.67%)	278(41.37%)	184(30.56%)	40(20.73%)	1(3.70%)	

Moderate	549(36.75%)	238(35.42%)	235(39.04%)	66(34.20%)	10(37.04%)	
Severe	390(26.10%)	118(17.56%)	172(28.57%)	84(43.52%)	16(59.26%)	
HGS, kg	22.38(8.93)	23.09(9.13)	22.33(8.72)	20.76(8.58)	17.79(8.61)	<0.001
EORTCQLQ-C30	39.21(4.79)	39.13(4.88)	39.44(4.57)	38.96(4.99)	38.03(5.72)	0.294
PN, yes	188(12.58%)	71(10.57%)	72(11.96%)	40(20.73%)	5(18.52%)	0.002
EN, yes	146(9.77%)	58(8.63%)	60(9.97%)	26(13.47%)	2(7.41%)	0.256

Values are mean (standard deviation) or n (%).

BMI: body mass index, ECOG: Eastern Cooperative Oncology Group, CONUT: Controlling Nutritional Status score, PNI: prognostic nutritional index, NRI: nutritional risk index, PG-SGA: patient-generated subjective global assessment, HGS: hand grip strength, EORTC QLQ-C30: European Organization for Research and Treatment of Cancer Quality of Life Questionnaire, PN: parenteral nutrition, EN: enteral nutrition.

Supplementary table 3. Baseline Characteristics of the Study Population stratified by NRI.

NRI	Overall<br>N=1494	Absent<br>N=617	Mild malnutrition<br>N=174	Moderate malnutrition<br>N=607	Severe malnutrition<br>N=96	*P*-value
Age, years	70.76(5.16)	70.12(4.86)	70.40(4.84)	71.31(5.35)	71.98(5.82)	<0.001
Gender, male	951(63.65%)	361(58.51%)	115(66.09%)	405(66.72%)	70(72.92%)	0.004
BMI, kg/m^2	22.58(3.55)	23.35(3.51)	23.01(3.36)	21.93(3.46)	20.89(3.46)	<0.001
Smoking, yes	696(46.59%)	264(42.79%)	86(49.43%)	294(48.43%)	52(54.17%)	0.066
Alcohol, yes	289(19.34%)	102(16.53%)	39(22.41%)	128(21.09%)	20(20.83%)	0.139
Comorbidities						
Absent	1081(72.36%)	448(72.61%)	126(72.41%)	435(71.66%)	72(75.00%)	
Hypertension	337(22.56%)	140(22.69%)	42(24.14%)	138(22.73%)	17(17.71%)	
Others	76(5.09%)	29(4.70%)	6(3.45%)	34(5.60%)	7(7.29%)	
Tumor location						<0.001
Lung	458(30.66%)	193(31.28%)	48(27.59%)	187(30.81%)	30(31.25%)	
Digestive	759(50.80%)	278(45.06%)	86(49.43%)	343(56.51%)	52(54.17%)	
Other	277(18.54%)	146(23.66%)	40(22.99%)	77(12.69%)	14(14.58%)	
Tumor stage:						<0.001
I	147(9.84%)	82(13.29%)	18(10.34%)	42(6.92%)	5(5.21%)	
II	336(22.49%)	157(25.45%)	32(18.39%)	128(21.09%)	19(19.79%)	
III	373(24.97%)	154(24.96%)	52(29.89%)	147(24.22%)	20(20.83%)	
IV	638(42.70%)	224(36.30%)	72(41.38%)	290(47.78%)	52(54.17%)	
Chemotherapy, yes	939(62.85%)	385(62.40%)	124(71.26%)	373(61.45%)	57(59.38%)	0.096
Immunotherapy, yes	109(7.30%)	51(8.27%)	13(7.47%)	41(6.75%)	4(4.17%)	0.472
Surgery, yes	357(23.90%)	144(23.34%)	33(18.97%)	150(24.71%)	30(31.25%)	0.138
ECOG	1.09(0.80)	0.98(0.71)	0.93(0.70)	1.18(0.82)	1.48(1.05)	<0.001
Albumin, g/dl	3.80(0.53)	4.26(0.26)	3.88(0.12)	3.50(0.25)	2.60(0.34)	<0.001
Cholesterol, mg/dl	182.95(60.94)	194.51(53.55)	187.73(60.15)	175.50(67.07)	147.07(43.74)	<0.001
Lymphocyte, 10^9/L	1.67(1.37)	1.76(1.04)	1.74(1.16)	1.62(1.75)	1.29(0.57)	0.010
CONUT, as continuous	2.30(2.21)	1.22(1.23)	1.43(1.27)	2.88(2.00)	7.19(1.84)	<0.001
Category						
Absent	672(44.98%)	409(66.29%)	101(58.05%)	162(26.69%)	0(0.00%)	
Mild	602(40.29%)	202(32.74%)	70(40.23%)	323(53.21%)	7(7.29%)	
Moderate	193(12.92%)	6(0.97%)	3(1.72%)	117(19.28%)	67(69.79%)	
Severe	27(1.81%)	0(0.00%)	0(0.00%)	5(0.82%)	22(22.92%)	
PNI, as continuous	46.05(9.90)	51.44(5.84)	47.49(5.88)	43.07(9.18)	27.70(11.33)	<0.001
Category						
Absent	1320(88.35%)	617(100.00%)	174(100.00%)	517(85.17%)	12(12.50%)	
Moderate	77(5.15%)	0(0.00%)	0(0.00%)	64(10.54%)	13(13.54%)	
Severe	97(6.49%)	0(0.00%)	0(0.00%)	26(4.28%)	71(73.96%)	
NRI, as continuous	96.96(10.32)	104.98(3.83)	98.78(0.70)	92.36(3.77)	71.20(16.03)	<0.001
Category						
Absent	617(41.30%)	617(100.00%)	0(0.00%)	0(0.00%)	0(0.00%)	
Mild	174(11.65%)	0(0.00%)	174(100.00%)	0(0.00%)	0(0.00%)	
Moderate	607(40.63%)	0(0.00%)	0(0.00%)	607(100.00%)	0(0.00%)	
Severe	96(6.43%)	0(0.00%)	0(0.00%)	0(0.00%)	96(100.00%)	
PG-SGA, as continuous	6.26(4.48)	4.67(3.32)	5.47(3.78)	7.53(4.79)	9.90(5.43)	<0.001
Category						
Absent	52(3.48%)	29(4.70%)	9(5.17%)	12(1.98%)	2(2.08%)	
Mild	503(33.67%)	282(45.71%)	63(36.21%)	145(23.89%)	13(13.54%)	

Moderate	549(36.75%)	229(37.12%)	65(37.36%)	227(37.40%)	28(29.17%)	
Severe	390(26.10%)	77(12.48%)	37(21.26%)	223(36.74%)	53(55.21%)	
HGS,kg	22.38(8.93)	23.25(9.11)	22.93(9.20)	21.74(8.53)	19.97(9.16)	0.001
EORTCQLQ-C30	39.21(4.79)	39.34(4.88)	39.28(4.10)	39.10(4.82)	39.01(5.26)	0.796
PN,yes	188(12.58%)	78(12.64%)	21(12.07%)	69(11.37%)	20(20.83%)	0.079
EN,yes	146(9.77%)	66(10.70%)	16(9.20%)	47(7.74%)	17(17.71%)	0.016

Values are mean (standard deviation) or n (%).

BMI: body mass index, ECOG: Eastern Cooperative Oncology Group, CONUT: Controlling Nutritional Status score, PNI: prognostic nutritional index, NRI: nutritional risk index, PG-SGA: patient-generated subjective global assessment, HGS: hand grip strength, EORTC QLQ-C30: European Organization for Research and Treatment of Cancer Quality of Life Questionnaire, PN: parenteral nutrition, EN: enteral nutrition.

Supplementary table 4. Baseline Characteristics of the Study Population stratified by PNI.

PNI	Overall N=1494	Absent N=672	Moderate malnutrition N=193	Severe malnutrition N=27	P-value
Age, years	70.76(5.16)	70.62(5.07)	71.43(5.61)	72.08(5.75)	0.013
Gender, male	951(63.65%)	826(62.58%)	54(70.13%)	71(73.20%)	0.053
BMI, kg/m^2	22.58(3.55)	22.75(3.52)	21.82(3.58)	20.86(3.42)	<0.001
Smoking, yes	696(46.59%)	609(46.14%)	36(46.75%)	51(52.58%)	0.471
Alcohol, yes	289(19.34%)	254(19.24%)	18(23.38%)	17(17.53%)	0.601
Comorbidities					
Absent	1081(72.36%)	954(72.27%)	60(77.92%)	67(69.07%)	
Hypertension	337(22.56%)	307(23.26%)	10(12.99%)	20(20.62%)	
Others	76(5.09%)	59(4.47%)	7(9.09%)	10(10.31%)	
Tumor location					0.104
Lung	458(30.66%)	411(31.14%)	16(20.78%)	31(31.96%)	
Digestive	759(50.80%)	657(49.77%)	48(62.34%)	54(55.67%)	
Other	277(18.54%)	252(19.09%)	13(16.88%)	12(12.37%)	
Tumor stage:					0.054
I	147(9.84%)	135(10.23%)	6(7.79%)	6(6.19%)	
II	336(22.49%)	303(22.95%)	11(14.29%)	22(22.68%)	
III	373(24.97%)	338(25.61%)	17(22.08%)	18(18.56%)	
IV	638(42.70%)	544(41.21%)	43(55.84%)	51(52.58%)	
Chemotherapy, yes	939(62.85%)	848(64.24%)	37(48.05%)	54(55.67%)	0.005
Immunotherapy, yes	109(7.30%)	96(7.27%)	7(9.09%)	6(6.19%)	0.762
Surgery, yes	357(23.90%)	306(23.18%)	25(32.47%)	26(26.80%)	0.14
ECOG	1.09(0.80)	1.05(0.76)	1.29(0.87)	1.45(1.04)	<0.001
Albumin, g/dl	3.80(0.53)	3.93(0.40)	3.11(0.22)	2.64(0.36)	<0.001
Cholesterol, mg/dl	182.95(60.94)	186.97(61.03)	159.55(55.14)	146.72(46.65)	<0.001
Lymphocyte, 10^9/L	1.67(1.37)	1.75(1.42)	1.10(0.44)	1.00(0.48)	<0.001
CONUT, as continuous	2.30(2.21)	1.73(1.51)	5.42(1.18)	7.65(1.50)	<0.001
Category					
Absent	672(44.98%)	672(50.91%)	0(0.00%)	0(0.00%)	
Mild	602(40.29%)	579(43.86%)	22(28.57%)	1(1.03%)	
Moderate	193(12.92%)	69(5.23%)	55(71.43%)	69(71.13%)	
Severe	27(1.81%)	0(0.00%)	0(0.00%)	27(27.84%)	
PNI,as continuous	46.05(9.90)	48.03(8.14)	36.64(0.84)	26.63(10.35)	<0.001
Category					
Absent	1320(88.35%)	1320(100.00%)	0(0.00%)	0(0.00%)	
Moderate	77(5.15%)	0(0.00%)	77(100.00%)	0(0.00%)	
Severe	97(6.49%)	0(0.00%)	0(0.00%)	97(100.00%)	
NRI,as continuous	96.96(10.32)	99.34(6.60)	86.72(3.96)	72.67(16.92)	<0.001
Category					
Absent	617(41.30%)	617(46.74%)	0(0.00%)	0(0.00%)	
Mild	174(11.65%)	174(13.18%)	0(0.00%)	0(0.00%)	
Moderate	607(40.63%)	517(39.17%)	64(83.12%)	26(26.80%)	
Severe	96(6.43%)	12(0.91%)	13(16.88%)	71(73.20%)	
PG-SGA, as continuous	6.26(4.48)	5.94(4.24)	8.16(5.13)	9.15(5.54)	<0.001
Category					
Absent	52(3.48%)	49(3.71%)	1(1.30%)	2(2.06%)	
Mild	503(33.67%)	473(35.83%)	14(18.18%)	16(16.49%)	

Moderate	549(36.75%)	488(36.97%)	29(37.66%)	32(32.99%)	
Severe	390(26.10%)	310(23.48%)	33(42.86%)	47(48.45%)	
HGS, kg	22.38(8.93)	22.65(8.93)	20.67(8.69)	20.09(8.79)	0.005
EORTCQLQ-C30	39.21(4.79)	39.28(4.78)	39.27(4.67)	38.33(5.00)	0.170
PN, yes	188(12.58%)	153(11.59%)	13(16.88%)	22(22.68%)	0.003
EN, yes	146(9.77%)	125(9.47%)	5(6.49%)	16(16.49%)	0.049

Values are mean (standard deviation) or n (%).

BMI: body mass index, ECOG: Eastern Cooperative Oncology Group, CONUT: Controlling Nutritional Status score, PNI: prognostic nutritional index, NRI: nutritional risk index, PG-SGA: patient-generated subjective global assessment, HGS: hand grip strength, EORTC QLQ-C30: European Organization for Research and Treatment of Cancer Quality of Life Questionnaire, PN: parenteral nutrition, EN: enteral nutrition.

Supplementary table 5. Kappa agreement for CONUT, PNI, NRI index

	CONUT vs. PNI	CONUT vs. NRI	NRI vs. PNI
Cohen's kappa statistic (95% CI)	0.20 (0.17-0.22)	0.36 (0.31-0.41)	0.17 (0.14-0.20)
P-value	<0.001	<0.001	<0.001

Supplementary table 6. Sensitivity, specificity, positive predictive value and negative predictive value for CONUT, PNI, NRI index compared with PG-SGA

Variables	AUC	Sensitivity	Specificity	Positive predictive value	Negative predictive value	Cohen's kappa statistic
		(95% CI)	(95% CI)	(95% CI)	(95% CI)	(95% CI)
CONUT	0.595	0.62 (0.59-0.65)	0.57 (0.53-0.61)	0.71 (0.67-0.74)	0.47 (0.44-0.51)	0.18 (0.13-0.23)*
PNI	0.545	0.15 (0.13-0.17)	0.94 (0.92-0.96)	0.81 (0.75-0.84)	0.40 (0.35-0.49)	0.07 (0.05-0.09)*
NRI	0.617	0.67 (0.64-0.70)	0.56 (0.52-0.60)	0.72 (0.69-0.75)	0.50 (0.47-0.55)	0.23 (0.18-0.28)*

*P<0.001

Supplementary table 7. Univariable Cox regression analyses of factors predicting all-cause mortality.

Characteristic	HR(95%CI)	*P*-value
Age, per SD	1.35(1.17-1.55)	<0.001
Gender		
Male	Ref.	Ref.
Female	0.64(0.54-0.76)	<0.001
BMI	0.93(0.91-0.95)	<0.001
Smoking		
No	Ref.	Ref.
Yes	1.32(1.13-1.54)	0.001
Alcohol		
No	Ref.	Ref.
Yes	1.19(0.98-1.44)	0.073
Comorbidities		
Absent	Ref.	Ref.
Hypertension	1.04(0.86-1.25)	0.713
Others	1.02(0.72-1.45)	0.896
Tumor location		
Lung	Ref.	Ref.
Digestive	0.80(0.67-0.94)	0.007
Other	0.34(0.25-0.45)	<0.001
Tumor stage:		
I	Ref.	Ref.
II	1.88(1.11-3.19)	0.019
III	3.53(2.13-5.85)	<0.001
IV	9.47(5.83-15.4)	<0.001
Chemotherapy		
No	Ref.	Ref.
Yes	1.48(1.25-1.76)	<0.001
Immunotherapy		
No	Ref.	Ref.
Yes	1.09(0.82-1.45)	0.548
Surgery		
No	Ref.	Ref.
Yes	0.47(0.38-0.58)	<0.001
ECOG	1.50(1.38-1.64)	<0.001
Albumin	0.48(0.42-0.55)	<0.001
Cholesterol	1.00(1.00-1.00)	0.247
Lymphocyte	0.90(0.80-1.00)	0.045
CONUT, as continuous	1.16(1.12-1.19)	<0.001
Category		
Absent	Ref.	Ref.
Mild	1.64(1.37-1.95)	<0.001
Moderate	2.48(1.97-3.11)	<0.001
Severe	3.48(2.18-5.56)	<0.001
NRI, as continuous	0.98(0.97-0.98)	<0.001
Category		
Absent	Ref.	Ref.
Mild	1.34(1.01-1.78)	0.044

Moderate	2.23(1.85-2.67)	<0.001
Severe	3.70(2.77-4.95)	<0.001
PNI, as continuous	0.97(0.96-0.98)	<0.001
Category		
Absent	Ref.	Ref.
Moderate	1.91(1.41-2.59)	<0.001
Severe	2.58(1.99-3.34)	<0.001
PG-SGA, as continuous	1.09(1.07-1.11)	<0.001
Category		
Absent	Ref.	Ref.
Mild	1.11(0.65-1.88)	0.702
Moderate	1.54(0.91-2.60)	0.105
Severe	2.79(1.66-4.71)	<0.001
HGS	0.99(0.98-1.00)	0.006
EORTC QLQ-C30	0.93(0.85-1.01)	0.076
PN		
No	Ref.	Ref.
Yes	1.01(0.78-1.29)	0.967
EN		
No	Ref.	Ref.
Yes	0.73(0.54-0.99)	0.043

BMI: body mass index, ECOG: Eastern Cooperative Oncology Group, CONUT: Controlling Nutritional Status score, PNI: prognostic nutritional index, NRI: nutritional risk index, PG-SGA: patient-generated subjective global assessment, HGS: hand grip strength, EORTC QLQ-C30: European Organization for Research and Treatment of Cancer Quality of Life Questionnaire, PN: parenteral nutrition, EN: enteral nutrition.

Supplementary table 8. Multivariate Cox proportional hazards analyses of malnutrition indexes to predict all-cause mortality according to the location of cancer.

	crude HR(95%CI)	*P*-value	adjusted HR (95% CI)[a]	*P*-value	adjusted HR(95%CI)[b]	*P*-value
**Lung**						
CONUT, as continuous	1.15(1.09-1.22)	<0.001	1.12(1.06-1.18)	<0.001	1.09(1.02-1.15)	0.007
Category						
Absent	Ref.		Ref.		Ref.	
Mild	1.66(1.25-2.21)	<0.001	1.53(1.14-2.04)	0.004	1.42(1.05-1.92)	0.022
Moderate	2.05(1.36-3.07)	<0.001	1.62(1.07-2.47)	0.024	1.29(0.83-2.01)	0.251
Severe	4.59(2.11-9.97)	<0.001	3.59(1.64-7.86)	0.001	2.78(1.22-6.34)	0.015
PNI, as continuous	0.98(0.97-0.99)	<0.001	0.98(0.97-1.00)	0.024	0.99(0.97-1.00)	0.080
Category						
Absent	Ref.		Ref.		Ref.	
Moderate	1.58(0.86-2.9)	0.138	1.34(0.73-2.48)	0.344	1.49(0.80-2.79)	0.208
Severe	2.36(1.5-3.7)	<0.001	2.13(1.35-3.35)	0.001	2.15(1.34-3.47)	0.002
NRI, as continuous	0.98(0.97-0.99)	<0.001	0.98(0.98-0.99)	0.001	0.99(0.98-1.00)	0.011
Category						
Absent	Ref.		Ref.		Ref.	
Mild	1.03(0.63-1.68)	0.910	0.94(0.58-1.55)	0.818	1.05(0.64-1.74)	0.836
Moderate	1.92(1.44-2.56)	<0.001	1.57(1.16-2.13)	0.003	1.37(1.00-1.86)	0.047
Severe	2.57(1.55-4.25)	<0.001	2.15(1.29-3.59)	0.003	2.19(1.28-3.73)	0.004
**Digestive**						
CONUT, as continuous	1.11(1.06-1.16)	<0.001	1.1(1.05-1.15)	<0.001	1.07(1.02-1.12)	0.004
Category						
Absent	Ref.		Ref.		Ref.	
Mild	1.35(1.06-1.73)	0.015	1.33(1.04-1.70)	0.021	1.31(1.02-1.69)	0.034
Moderate	2.07(1.54-2.79)	<0.001	1.91(1.41-2.59)	<0.001	1.97(1.42-2.74)	<0.001
Severe	2.17(1.1-4.28)	0.025	1.96(0.99-3.88)	0.052	1.28(0.61-2.68)	0.510
PNI, as continuous	0.97(0.96-0.98)	<0.001	0.97(0.96-0.99)	<0.001	0.98(0.97-0.99)	0.002
Category						
Absent	Ref.		Ref.		Ref.	
Moderate	1.75(1.18-2.59)	0.006	1.62(1.09-2.42)	0.018	1.50(0.98-2.28)	0.061
Severe	2.27(1.6-3.21)	<0.001	2.06(1.45-2.94)	<0.001	1.91(1.31-2.79)	<0.001
NRI, as continuous	0.97(0.96-0.98)	<0.001	0.97(0.97-0.98)	<0.001	0.98(0.97-0.99)	<0.001
Category						
Absent	Ref.		Ref.		Ref.	
Mild	1.82(1.25-2.65)	0.002	1.81(1.24-2.63)	0.002	1.77(1.21-2.58)	0.003
Moderate	2.09(1.61-2.7)	<0.001	2.01(1.55-2.61)	<0.001	2.01(1.54-2.62)	<0.001
Severe	3.68(2.47-5.48)	<0.001	3.34(2.22-5.03)	<0.001	2.89(1.85-4.52)	<0.001
**Other**						
CONUT, as continuous	1.31(1.21-1.42)	<0.001	1.31(1.2-1.43)	<0.001	1.38(1.23-1.56)	<0.001
Category						
Absent	Ref.		Ref.		Ref.	
Mild	2.77(1.51-5.08)	0.001	2.27(1.21-4.26)	0.01	1.85(0.92-3.70)	0.082
Moderate	7.19(3.36-15.39)	<0.001	5.52(2.53-12.02)	<0.001	5.35(2.01-14.20)	<0.001
Severe	12.85(3.75-43.98)	<0.001	13.34(3.67-48.52)	<0.001	23.18(5.14-104.58)	<0.001
PNI, as continuous	0.95(0.93-0.97)	<0.001	0.96(0.93-0.98)	<0.001	0.95(0.93-0.98)	0.002
Category						
Absent	Ref.		Ref.		Ref.	

Moderate	4.67(2.1-10.38)	<0.001	5.08(2.26-11.42)	<0.001	4.3(1.72-10.76)	0.002
Severe	5.71(2.55-12.79)	<0.001	5.00(2.22-11.27)	<0.001	5.09(1.93-13.44)	0.001
NRI, as continuous	0.97(0.96-0.99)	<0.001	0.97(0.96-0.99)	0.001	0.98(0.95-1.00)	0.023
Category						
Absent	Ref.		Ref.		Ref.	
Mild	0.87(0.32-2.32)	0.778	0.78(0.29-2.10)	0.629	0.54(0.20-1.49)	0.235
Moderate	3.28(1.81-5.94)	<0.001	2.81(1.54-5.12)	<0.001	2.04(1.03-4.03)	0.041
Severe	8.33(3.61-19.2)	<0.001	6.86(2.94-16.02)	<0.001	5.55(2.15-14.31)	<0.001

a: Adjusted by age, gender, BMI

b: Adjusted by age, gender, BMI, comorbidities disease, smoking, alcohol, tumor location, tumor stage, chemotherapy, immunotherapy, surgery, parenteral nutrition intervention, enteral nutrition intervention, ECOG, hand grip strength, EORTC QLQ-C30. For NRI, total cholesterol and lymphocyte count were adjusted additionally. For PNI, lymphocyte count was adjusted additionally.

HR: hazard ratio, CI: confidence interval, ECOG: Eastern Cooperative Oncology Group, CONUT: Controlling Nutritional Status score, PNI: prognostic nutritional index, NRI: nutritional risk index, PG-SGA: patient-generated subjective global assessment, HGS: hand grip strength, EORTC QLQ-C30: European Organization for Research and Treatment of Cancer Quality of Life Questionnaire

Supplementary table 9. Multivariate Cox proportional hazards analyses of malnutrition indexes to predict all-cause mortality according to exclude patients died within 6 months.

	crude HR(95%CI)	*P*-value	adjusted HR (95% CI)[a]	*P*-value	adjusted HR(95%CI)[b]	*P*-value
CONUT, as continuous	1.11(1.07-1.16)	<0.001	1.09(1.04-1.13)	<0.001	1.06(1.02-1.11)	0.007
Category						
Absent	Ref.				Ref.	
Mild	1.49(1.21-1.84)	<0.001	1.40(1.14-1.73)	0.001	1.29(1.04-1.60)	0.021
Moderate	1.88(1.40-2.53)	<0.001	1.60(1.18-2.16)	0.002	1.47(1.07-2.02)	0.016
Severe	2.35(1.20-4.59)	0.013	1.97(1.00-3.86)	0.050	1.50(0.73-3.06)	0.270
PNI, as continuous	0.97(0.96-0.98)	<0.001	0.98(0.97-0.99)	<0.001	0.98(0.97-1.00)	0.005
Category						
Absent	Ref.					
Moderate	1.44(0.95-2.20)	0.089	1.33(0.87-2.03)	0.190	1.29(0.84-2.00)	0.244
Severe	1.89(1.30-2.74)	<0.001	1.63(1.12-2.37)	0.011	1.66(1.12-2.45)	0.011
NRI, as continuous	0.98(0.97-0.99)	<0.001	0.98(0.97-0.99)	<0.001	0.98(0.98-0.99)	<0.001
Category						
Absent	Ref.					
Mild	1.27(0.91-1.77)	0.155	1.22(0.88-1.70)	0.231	1.23(0.88-1.71)	0.229
Moderate	2.01(1.62-2.49)	<0.001	1.82(1.47-2.27)	<0.001	1.63(1.30-2.03)	<0.001
Severe	2.62(1.76-3.91)	<0.001	2.22(1.48-3.33)	<0.001	2.10(1.37-3.22)	<0.001

a: Adjusted by age, gender, BMI

b: Adjusted by age, gender, BMI, comorbidities disease, smoking, alcohol, tumor location, tumor stage, chemotherapy, immunotherapy, surgery, parenteral nutrition intervention, enteral nutrition intervention, ECOG, hand grip strength, EORTC QLQ-C30. For NRI, total cholesterol and lymphocyte count were adjusted additionally. For PNI, lymphocyte count was adjusted additionally.

HR: hazard ratio, CI: confidence interval, ECOG: Eastern Cooperative Oncology Group, CONUT: Controlling Nutritional Status score, PNI: prognostic nutritional index, NRI: nutritional risk index, PG-SGA: patient-generated subjective global assessment, HGS: hand grip strength, EORTC QLQ-C30: European Organization for Research and Treatment of Cancer Quality of Life Questionnaire

Supplementary table 10. Each parameters of EORTC QLQ-C30 stratified by CONUT.

CONUT	Overall N=1494	Absent N=672	Mild N=602	Moderate N=193	Severe N=27	*P*-value
Physical function	86.7 (73.3-100)	93.3 (80.0-100)	86.7 (73.3-100)	80.0 (66.7-93.3)	80.0 (53.3-86.7)	<0.001
Role function	83.3 (66.7-100)	100 (66.7-100)	83.3 (66.7-100)	66.7 (66.7-100)	66.7 (33.3-100)	<0.001
Emotional function	100 (75.0-100)	100 (83.3-100)	95.8 (75.0-100)	100 (75.0-100)	91.7 (70.8-95.8)	0.084
Congnitive function	100 (83.3-100)	100 (83.3-100)	83.3 (83.3-100)	83.3 (66.7-100)	83.3 (66.7-100)	<0.001
Social function	66.7 (66.7-100)	83.3 (66.7-100)	66.7 (66.7-100)	66.7 (66.7-100)	66.7 (58.3-100)	<0.001
Global QOL	66.7 (50.0-75.0)	66.7 (50.0-83.3)	58.3 (50.0-66.7)	50.0 (50.0-66.7)	50.0 (33.3-66.7)	<0.001
Fatigue	11.1 (0.00-33.3)	11.1 (0.00-22.2)	22.2 (0.00-33.3)	22.2 (0.00-33.3)	33.3 (11.1-55.6)	<0.001
Nausea and vomiting	0.00 (0.00-0.00)	0.00 (0.00-0.00)	0.00 (0.00-0.00)	0.00 (0.00-0.00)	0.00 (0.00-0.00)	<0.001
Pain	0.00 (0.00-16.7)	0.00 (0.00-16.7)	0.00 (0.00-16.7)	0.00 (0.00-33.3)	0.00 (0.00-33.3)	<0.001
Dyspnea	0.00 (0.00-33.3)	0.00 (0.00-8.33)	0.00 (0.00-0.00)	0.00 (0.00-33.3)	0.00 (0.00-33.3)	0.008
Sleep disturbance	0.00 (0.00-33.3)	0.00 (0.00-33.3)	0.00 (0.00-33.3)	33.3 (0.00-33.3)	0.00 (0.00-50.0)	0.004
Appetite loss	0.00 (0.00-33.3)	0.00 (0.00-0.00)	0.00 (0.00-33.3)	0.00 (0.00-33.3)	0.00 (0.00-50.0)	<0.001
Constipation	0.00 (0.00-0.00)	0.00 (0.00-0.00)	0.00 (0.00-0.00)	0.00 (0.00-0.00)	0.00 (0.00-0.00)	0.422
Diarrhea	0.00 (0.00-0.00)	0.00 (0.00-0.00)	0.00 (0.00-0.00)	0.00 (0.00-0.00)	0.00 (0.00-0.00)	0.33
Financial difficulties	33.3 (0.00-33.3)	33.3 (0.00-33.3)	33.3 (0.00-33.3)	33.3 (0.00-33.3)	33.3 (33.3-33.3)	<0.001
overall	39.21 (4.79)	39.13 (4.88)	39.44 (4.57)	38.96 (4.99)	38.03 (5.72)	0.294

Data are represented as median (interquartile range) .

Supplementary table 11. Each parameters of EORTC QLQ-C30 stratified by NRI.

NRI	Overall N=1494	Absent N=617	Mild N=174	Moderate N=607	Severe N=96	*P*-value
Physical function	86.7 (73.3-100)	93.3 (80.0-100)	93.3 (80.0-100)	86.7 (73.3-100)	80.0 (53.3-86.7)	<0.001
Role function	83.3 (66.7-100)	100 (66.7-100)	100 (66.7-100)	66.7 (66.7-100)	66.7 (50.0-100)	<0.001
Emotional function	100 (75.0-100)	100 (83.3-100)	100 (83.3-100)	100 (75.0-100)	83.3 (66.7-100)	0.001
Congnitive function	100 (83.3-100)	100 (83.3-100)	100 (83.3-100)	83.3 (66.7-100)	83.3 (66.7-100)	<0.001
Social function	66.7 (66.7-100)	83.3 (66.7-100)	100 (66.7-100)	66.7 (66.7-100)	66.7 (33.3-100)	<0.001
Global QOL	66.7 (50.0-75.0)	66.7 (50.0-83.3)	66.7 (50.0-75.0)	58.3 (50.0-66.7)	50.0 (33.3-66.7)	<0.001
Fatigue	11.1 (0.00-33.3)	11.1 (0.00-33.3)	11.1 (0.00-22.2)	22.2 (0.00-33.3)	33.3 (11.1-44.4)	<0.001
Nausea and vomiting	0.00 (0.00-0.00)	0.00 (0.00-0.00)	0.00 (0.00-0.00)	0.00 (0.00-0.00)	0.00 (0.00-0.00)	0.001
Pain	0.00 (0.00-16.7)	0.00 (0.00-16.7)	0.00 (0.00-16.7)	0.00 (0.00-16.7)	0.00 (0.00-33.3)	0.003
Dyspnea	0.00 (0.00-33.3)	0.00 (0.00-0.00)	0.00 (0.00-25.0)	0.00 (0.00-33.3)	0.00 (0.00-33.3)	0.017
Sleep disturbance	0.00 (0.00-33.3)	0.00 (0.00-33.3)	0.00 (0.00-33.3)	0.00 (0.00-33.3)	33.3 (0.00-33.3)	0.003
Appetite loss	0.00 (0.00-33.3)	0.00 (0.00-0.00)	0.00 (0.00-25.0)	0.00 (0.00-33.3)	0.00 (0.00-33.3)	<0.001
Constipation	0.00 (0.00-0.00)	0.00 (0.00-0.00)	0.00 (0.00-0.00)	0.00 (0.00-0.00)	0.00 (0.00-8.33)	0.736
Diarrhea	0.00 (0.00-0.00)	0.00 (0.00-0.00)	0.00 (0.00-0.00)	0.00 (0.00-0.00)	0.00 (0.00-0.00)	0.546
Financial difficulties	33.3 (0.00-33.3)	33.3 (0.00-33.3)	33.3 (0.00-33.3)	33.3 (0.00-33.3)	33.3 (33.3-66.7)	<0.001
overall	39.21 (4.79)	39.34 (4.88)	39.28 (4.10)	39.10 (4.82)	39.01 (5.26)	0.796

Data are represented as median (interquartile range) .

Supplementary table 12. Each parameters of EORTC QLQ-C30 stratified by PNI .

PNI	Overall N=1494	Absent N=1320	Moderate N=77	Severe N=97	*P*-value
Physical function	86.7 (73.3-100)	86.7 (80.0-100)	73.3 (60.0-86.7)	80.0 (53.3-86.7)	<0.001
Role function	83.3 (66.7-100)	100 (66.7-100)	66.7 (66.7-100)	66.7 (66.7-100)	<0.001
Emotional function	100 (75.0-100)	100 (83.3-100)	100 (75.0-100)	91.7 (66.7-100)	0.076
Congnitive function	100 (83.3-100)	100 (83.3-100)	83.3 (66.7-100)	83.3 (66.7-100)	<0.001
Social function	66.7 (66.7-100)	66.7 (66.7-100)	66.7 (66.7-100)	66.7 (50.0-100)	<0.001
Global QOL	66.7 (50.0-75.0)	66.7 (50.0-75.0)	50.0 (33.3-66.7)	50.0 (33.3-66.7)	<0.001
Fatigue	11.1 (0.00-33.3)	11.1 (0.00-33.3)	22.2 (11.1-33.3)	33.3 (0.00-44.4)	<0.001
Nausea and vomiting	0.00 (0.00-0.00)	0.00 (0.00-0.00)	0.00 (0.00-0.00)	0.00 (0.00-0.00)	0.018
Pain	0.00 (0.00-16.7)	0.00 (0.00-16.7)	0.00 (0.00-33.3)	0.00 (0.00-33.3)	0.032
Dyspnea	0.00 (0.00-33.3)	0.00 (0.00-33.3)	0.00 (0.00-33.3)	0.00 (0.00-33.3)	0.07
Sleep disturbance	0.00 (0.00-33.3)	0.00 (0.00-33.3)	33.3 (0.00-33.3)	33.3 (0.00-33.3)	<0.001
Appetite loss	0.00 (0.00-33.3)	0.00 (0.00-33.3)	0.00 (0.00-33.3)	0.00 (0.00-33.3)	<0.001
Constipation	0.00 (0.00-0.00)	0.00 (0.00-0.00)	0.00 (0.00-0.00)	0.00 (0.00-0.00)	0.473
Diarrhea	0.00 (0.00-0.00)	0.00 (0.00-0.00)	0.00 (0.00-0.00)	0.00 (0.00-0.00)	0.787
Financial difficulties	33.3 (0.00-33.3)	33.3 (0.00-33.3)	33.3 (0.00-33.3)	33.3 (0.00-33.3)	0.009
overall	39.21 (4.79)	39.28 (4.78)	39.27 (4.67)	38.33 (5.00)	0.17

Data are represented as median (interquartile range) .

Supplementary table 13. Each parameters of EORTC QLQ-C30(with ECOG less than 2) stratified by CONUT

CONUT	Overall N=1299	Absent N=627	Mild N=511	Moderate N=144	Severe N=17	*P*-value
Physical function	86.7 (80.0-100)	93.3 (80.0-100)	86.7 (80.0-100)	86.7 (73.3-100)	80.0 (73.3-93.3)	<0.001
Role function	100 (66.7-100)	100 (66.7-100)	100 (66.7-100)	66.7 (66.7-100)	100 (66.7-100)	<0.001
Emotional function	100 (83.3-100)	100 (83.3-100)	100 (83.3-100)	100 (83.3-100)	91.7 (83.3-100)	0.717
Congnitive function	100 (83.3-100)	100 (83.3-100)	100 (83.3-100)	83.3 (66.7-100)	83.3 (83.3-100)	0.029
Social function	83.3 (66.7-100)	83.3 (66.7-100)	66.7 (66.7-100)	66.7 (66.7-100)	100 (66.7-100)	0.007
Global QOL	66.7 (50.0-75.0)	66.7 (50.0-83.3)	66.7 (50.0-75.0)	50.0 (50.0-66.7)	50.0 (33.3-66.7)	<0.001
Fatigue	11.1 (0.00-33.3)	11.1 (0.00-22.2)	11.1 (0.00-33.3)	22.2 (0.00-33.3)	22.2 (11.1-44.4)	<0.001
Nausea and vomiting	0.00 (0.00-0.00)	0.00 (0.00-0.00)	0.00 (0.00-0.00)	0.00 (0.00-0.00)	0.00 (0.00-0.00)	0.205
Pain	0.00 (0.00-16.7)	0.00 (0.00-16.7)	0.00 (0.00-16.7)	0.00 (0.00-16.7)	0.00 (0.00-33.3)	0.002
Dyspnea	0.00 (0.00-0.00)	0.00 (0.00-0.00)	0.00 (0.00-0.00)	0.00 (0.00-0.00)	0.00 (0.00-33.3)	0.373
Sleep disturbance	0.00 (0.00-33.3)	0.00 (0.00-33.3)	0.00 (0.00-33.3)	0.00 (0.00-33.3)	0.00 (0.00-33.3)	0.131
Appetite loss	0.00 (0.00-33.3)	0.00 (0.00-0.00)	0.00 (0.00-33.3)	0.00 (0.00-33.3)	0.00 (0.00-33.3)	<0.001
Constipation	0.00 (0.00-0.00)	0.00 (0.00-0.00)	0.00 (0.00-0.00)	0.00 (0.00-0.00)	0.00 (0.00-0.00)	0.84
Diarrhea	0.00 (0.00-0.00)	0.00 (0.00-0.00)	0.00 (0.00-0.00)	0.00 (0.00-0.00)	0.00 (0.00-0.00)	0.32
Financial difficulties	33.3 (0.00-33.3)	33.3 (0.00-33.3)	33.3 (0.00-33.3)	33.3 (0.00-33.3)	33.3 (33.3-33.3)	0.026

Data are represented as median (interquartile range) .

Supplementary table 14. Each parameters of EORTC QLQ-C30(with ECOG less than 2) stratified by NRI

NRI	Overall<br>N=1299	Absent<br>N=563	Mild<br>N=161	Moderate<br>N=507	Severe<br>N=68	*P*-value
Physical function	86.7 (80.0-100)	93.3 (86.7-100)	93.3 (86.7-100)	86.7 (80.0-100)	86.7 (73.3-93.3)	<0.001
Role function	100 (66.7-100)	100 (66.7-100)	100 (66.7-100)	83.3 (66.7-100)	66.7 (66.7-100)	<0.001
Emotional function	100 (83.3-100)	100 (83.3-100)	100 (83.3-100)	100 (83.3-100)	91.7 (72.9-100)	0.053
Congnitive function	100 (83.3-100)	100 (83.3-100)	100 (83.3-100)	83.3 (83.3-100)	83.3 (66.7-100)	0.001
Social function	83.3 (66.7-100)	83.3 (66.7-100)	100 (66.7-100)	66.7 (66.7-100)	66.7 (66.7-100)	<0.001
Global QOL	66.7 (50.0-75.0)	66.7 (50.0-83.3)	66.7 (50.0-75.0)	66.7 (50.0-66.7)	58.3 (50.0-66.7)	<0.001
Fatigue	11.1 (0.00-33.3)	11.1 (0.00-22.2)	0.00 (0.00-22.2)	22.2 (0.00-33.3)	22.2 (0.00-33.3)	<0.001
Nausea and vomiting	0.00 (0.00-0.00)	0.00 (0.00-0.00)	0.00 (0.00-0.00)	0.00 (0.00-0.00)	0.00 (0.00-0.00)	0.051
Pain	0.00 (0.00-16.7)	0.00 (0.00-16.7)	0.00 (0.00-16.7)	0.00 (0.00-16.7)	0.00 (0.00-33.3)	0.041
Dyspnea	0.00 (0.00-0.00)	0.00 (0.00-0.00)	0.00 (0.00-0.00)	0.00 (0.00-0.00)	0.00 (0.00-33.3)	0.813
Sleep disturbance	0.00 (0.00-33.3)	0.00 (0.00-33.3)	0.00 (0.00-33.3)	0.00 (0.00-33.3)	0.00 (0.00-33.3)	0.103
Appetite loss	0.00 (0.00-33.3)	0.00 (0.00-0.00)	0.00 (0.00-0.00)	0.00 (0.00-33.3)	0.00 (0.00-33.3)	<0.001
Constipation	0.00 (0.00-0.00)	0.00 (0.00-0.00)	0.00 (0.00-0.00)	0.00 (0.00-0.00)	0.00 (0.00-0.00)	0.97
Diarrhea	0.00 (0.00-0.00)	0.00 (0.00-0.00)	0.00 (0.00-0.00)	0.00 (0.00-0.00)	0.00 (0.00-0.00)	0.316
Financial difficulties	33.3 (0.00-33.3)	33.3 (0.00-33.3)	33.3 (0.00-33.3)	33.3 (0.00-33.3)	33.3 (0.00-66.7)	0.001

Data are represented as median (interquartile range) .

Supplementary table 15. Each parameters of EORTC QLQ-C30(with ECOG less than 2) stratified by PNI

PNI	Overall<br>N=1299	Absent<br>N=1172	Moderate<br>N=59	Severe<br>N=68	*P*-value
Physical function	86.7 (80.0-100)	93.3 (80.0-100)	86.7 (73.3-93.3)	86.7 (73.3-95.0)	<0.001
Role function	100 (66.7-100)	100 (66.7-100)	66.7 (66.7-100)	66.7 (66.7-100)	<0.001
Emotional function	100 (83.3-100)	100 (83.3-100)	100 (83.3-100)	95.8 (83.3-100)	0.643
Congnitive function	100 (83.3-100)	100 (83.3-100)	100 (83.3-100)	83.3 (66.7-100)	0.019
Social function	83.3 (66.7-100)	83.3 (66.7-100)	66.7 (66.7-100)	66.7 (66.7-100)	0.09
Global QOL	66.7 (50.0-75.0)	66.7 (50.0-75.0)	50.0 (50.0-66.7)	58.3 (50.0-66.7)	<0.001
Fatigue	11.1 (0.00-33.3)	11.1 (0.00-33.3)	22.2 (5.56-33.3)	22.2 (0.00-33.3)	0.001
Nausea and vomiting	0.00 (0.00-0.00)	0.00 (0.00-0.00)	0.00 (0.00-0.00)	0.00 (0.00-0.00)	0.706
Pain	0.00 (0.00-16.7)	0.00 (0.00-16.7)	0.00 (0.00-16.7)	0.00 (0.00-16.7)	0.398
Dyspnea	0.00 (0.00-0.00)	0.00 (0.00-0.00)	0.00 (0.00-0.00)	0.00 (0.00-0.00)	0.784
Sleep disturbance	0.00 (0.00-33.3)	0.00 (0.00-33.3)	0.00 (0.00-33.3)	0.00 (0.00-33.3)	0.056
Appetite loss	0.00 (0.00-33.3)	0.00 (0.00-0.00)	0.00 (0.00-33.3)	0.00 (0.00-33.3)	0.007
Constipation	0.00 (0.00-0.00)	0.00 (0.00-0.00)	0.00 (0.00-0.00)	0.00 (0.00-0.00)	0.208
Diarrhea	0.00 (0.00-0.00)	0.00 (0.00-0.00)	0.00 (0.00-0.00)	0.00 (0.00-0.00)	0.842
Financial difficulties	33.3 (0.00-33.3)	33.3 (0.00-33.3)	33.3 (0.00-33.3)	33.3 (0.00-41.7)	0.099

Data are represented as median (interquartile range) .

Supplementary table 16. Hazard risk for all cause mortality in elder patients treated with immunotherapy.

	crude HR(95%CI)	*P*-value	adjusted HR (95% CI)[a]	*P*-value	adjusted HR(95%CI)[b]	*P*-value
**NRI**						
Absent	Ref					
Mild	2.13(0.82-5.56)	0.121	1.9(0.73-4.97)	0.189	1.95(0.56-6.71)	0.291
Moderate	3.75(1.97-7.16)	<0.001	3.77(1.96-7.23)	<0.001	2.14(1.00-4.59)	0.051
Severe	10.56(3.43-32.49)	<0.001	10.22(3.18-32.85)	<0.001	5.83(1.34-25.36)	0.019
**PNI**						
Absent	Ref					
Moderate	4.92(2.06-11.75)	<0.001	5.42(2.22-13.24)	<0.001	2.41(0.73-7.16)	0.124
Severe	5.56(2.33-13.27)	<0.001	4.93(2.01-12.06)	<0.001	3.87(1.21-10.27)	0.004
**CONUT**						
Absent	Ref					
Mild	2.02(1.06-3.83)	0.032	1.87(0.97-3.61)	0.060	1.57(0.72-3.40)	0.256
Moderate	7.12(3.28-15.46)	<0.001	5.97(2.62-13.61)	<0.001	2.85(0.92-8.83)	0.069
Severe	NA(NA-NA)	(NA)	NA(NA-NA)	(NA)	NA(NA-NA)	(NA)

a: Adjusted by age, gender, BMI

b: Adjusted by age, gender, BMI, comorbidities disease, smoking, alcohol, tumor location, tumor stage, chemotherapy, immunotherapy, surgery, parenteral nutrition intervention, enteral nutrition intervention, ECOG, hand grip strength, EORTC QLQ-C30. For NRI, total cholesterol and lymphocyte count were adjusted additionally. For PNI, lymphocyte count was adjusted additionally.

HR: hazard ratio, CI: confidence interval, CONUT: Controlling Nutritional Status score, PNI: prognostic nutritional index, NRI: nutritional risk index

Supplementary Figure 1.

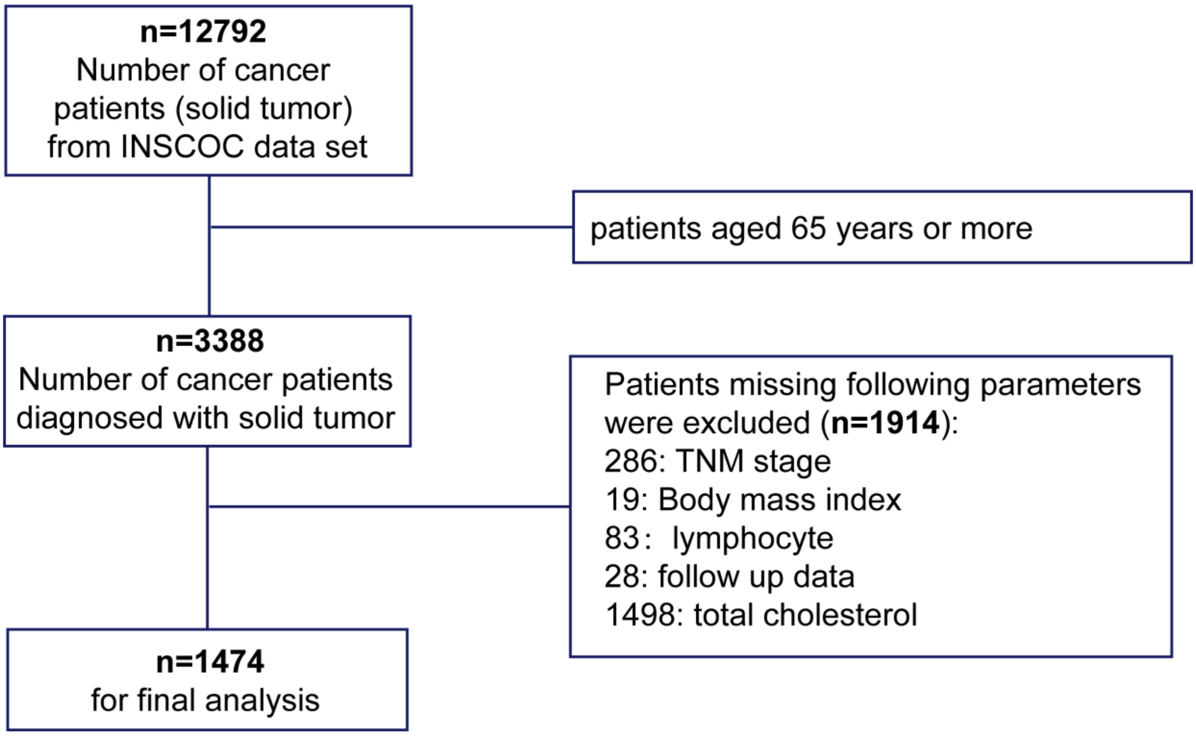

Supplementary Figure 2.

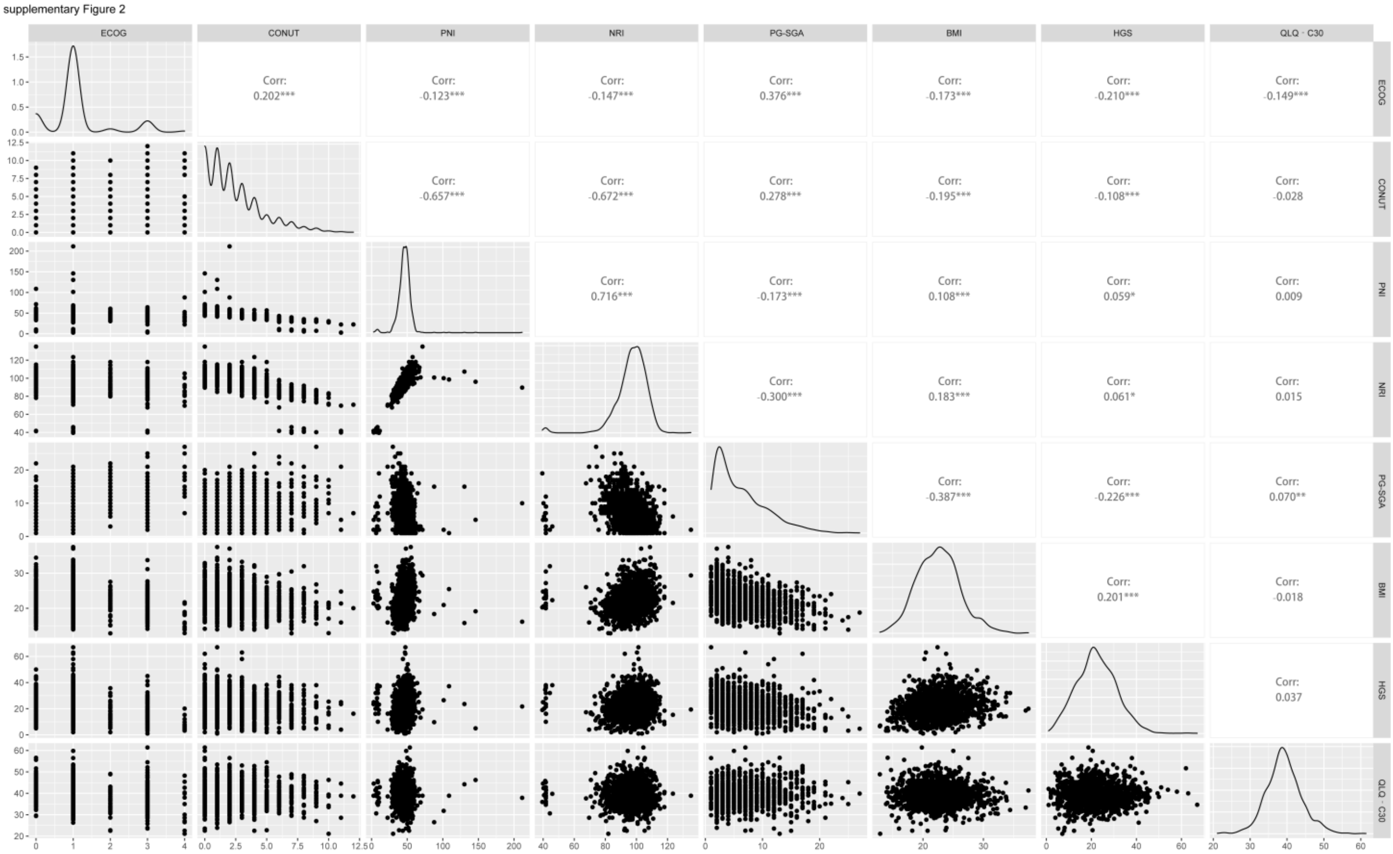

Correlation analysis of clinical parameters.

supplementary Figure 3

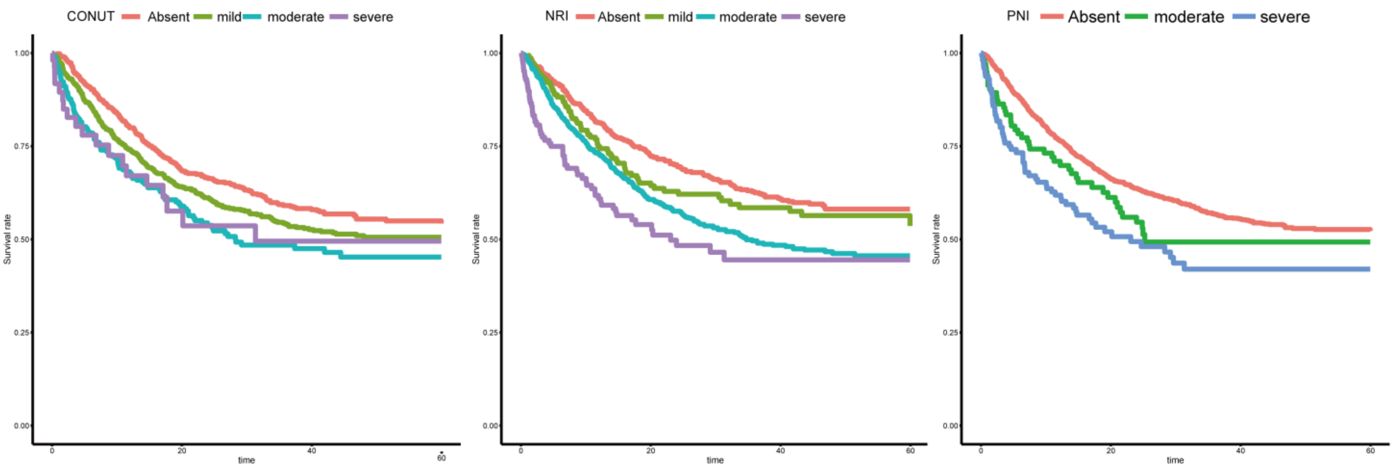

Adjusted Kaplan-Meier curves for all-cause mortality by the category of each malnutrition index in elderly patients with cancer. CONUT: Controlling Nutritional Status score, PNI: prognostic nutritional index, NRI: nutritional risk index. The value was adjusted for age, gender, body mass index, hypertension, other comorbidities disease, smoking, alcohol, tumor location, tumor stage, chemotherapy, immunotherapy, surgery, parenteral nutrition intervention, enteral nutrition intervention, ECOG, PG-SGA, hand grip strength, EORTC QLQ-C30. For NRI, total cholesterol and lymphocyte count were adjusted additionally. For PNI, lymphocyte count was adjusted additionally.

Supplementary Figure 4.

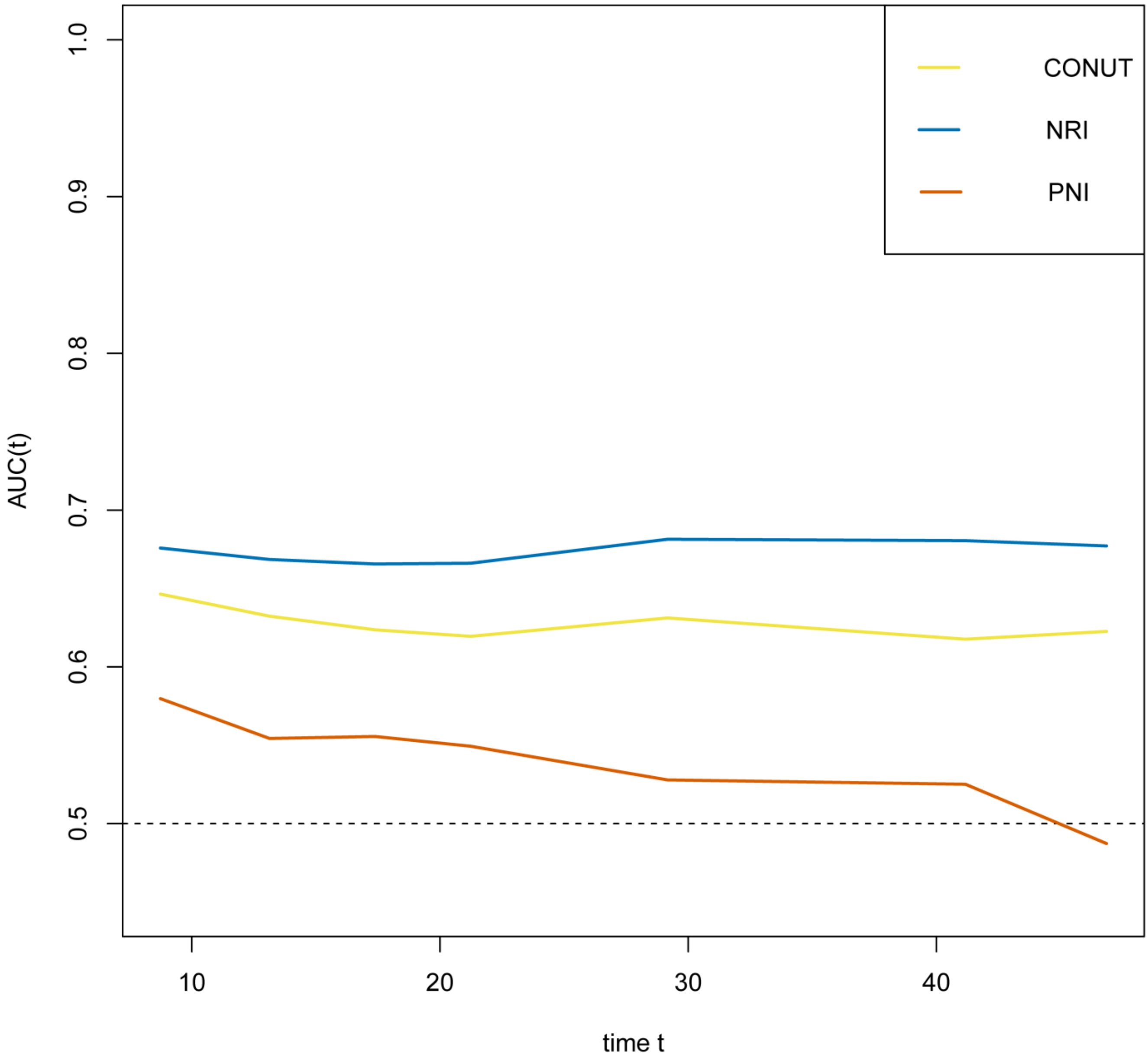

Supplementary Figure 5

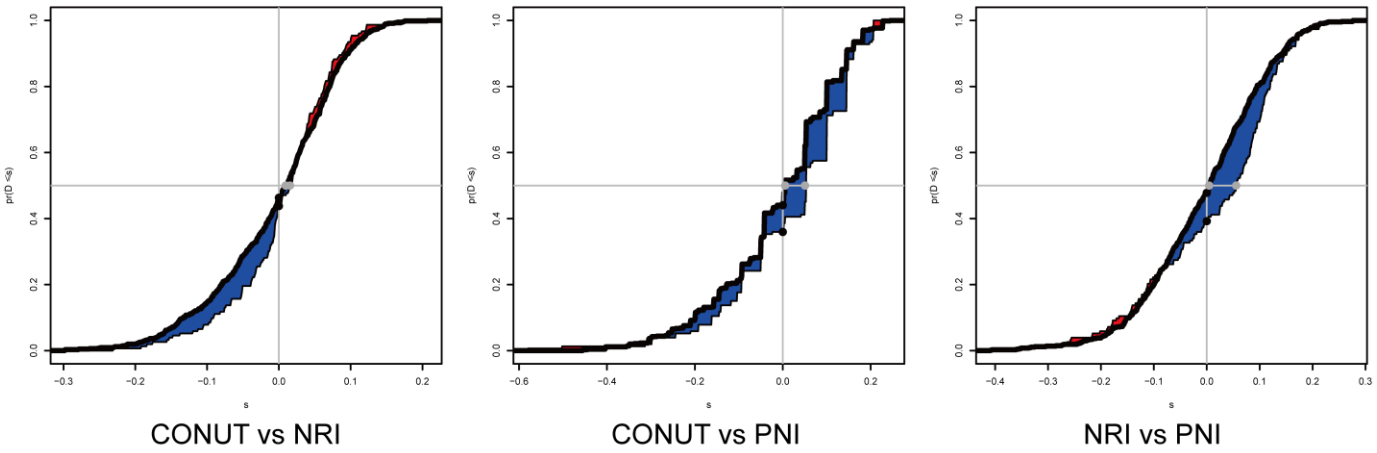

Plot to graphically display Integrated Discrimination Improvement (IDI), continuous Net Reclassification Improvement (NRI) and median improvement, for the additional value of malnutrition scores to TNM stage as assessed by the paired difference of risk scores.

Supplementary Figure 6.

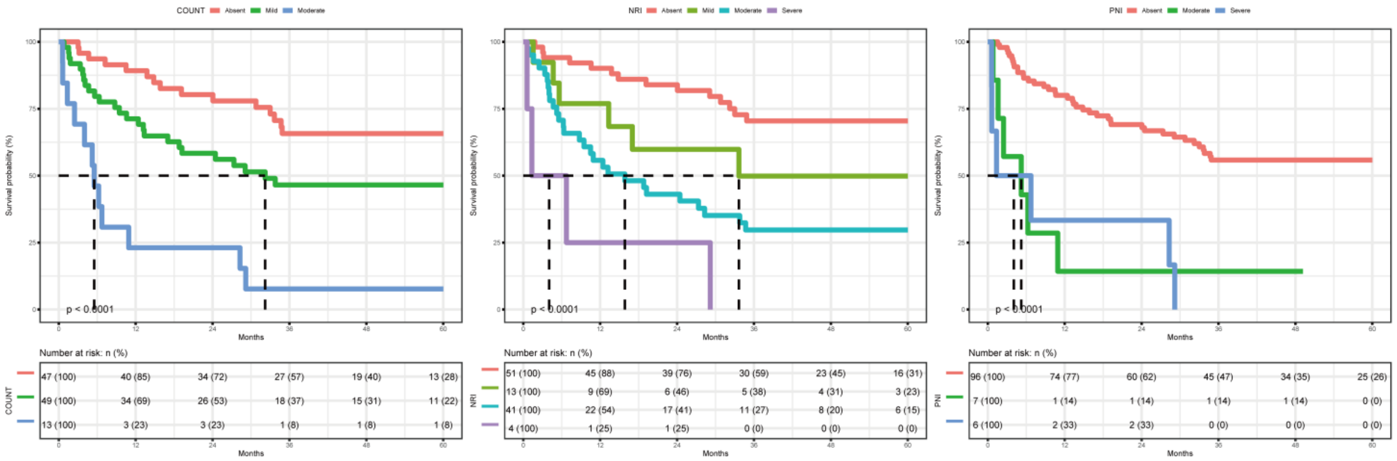

Supplement: Supplementary Table 1 — Procedures for the evaluation of each nutritional index. [file Data_Sheet_1.pdf]
